# Imaging-Based AI for Predicting Pathological Complete Response in Esophageal Cancer After Neoadjuvant Therapy: Systematic Review and Meta-Analysis

**DOI:** 10.2196/98520

**Published:** 2026-11-11

**Authors:** Zhiwei Li, Xin Gao, Jing Zhou, Lei Wang, Fuqiang Ren

**Affiliations:** 1 Department of Thoracic Surgery, Chengdu Sixth People’s Hospital, No. 369 Quanshui Road, Jinniu District, Chengdu, Sichuan, 610051, China, +86 18980017366

**Keywords:** artificial intelligence, pathological complete response, esophageal cancer, neoadjuvant therapy, meta-analysis

## Abstract

**Background:**

Imaging-based AI may help predict pathological complete response (pCR) after neoadjuvant therapy for esophageal cancer. However, an encouraging average diagnostic performance does not establish reliable performance across clinical settings or justify treatment decisions without further validation.

**Objective:**

We aimed to assess the predictive performance of imaging-based AI across neoadjuvant strategies, distinguish internal from external validation, and examine limitations affecting interpretation.

**Methods:**

PubMed, Embase, Web of Science, and the Cochrane Library were searched through August 7, 2026. Eligible English-language studies evaluated imaging-based AI against pathological assessment of pCR. PROBAST+AI (Prediction Model Risk of Bias Assessment Tool plus Artificial Intelligence) assessed study quality and risk of bias; GRADE (Grading of Recommendations Assessment, Development, and Evaluation) assessed certainty for sensitivity, specificity, and diagnostic odds ratio (DOR). Bivariate random-effects models synthesized validation groups separately. CIs described uncertainty in average performance, whereas 95% prediction intervals (PIs) described true performance in a similar new study.

**Results:**

Thirty-three studies contributed 27 internal-validation datasets with 1891 patients and 16 external-validation datasets with 777 patients. Ten publications contributed to both groups. The sum was 2668 validation observations; the unique patient count across datasets and publications could not be verified. Internal sensitivity was 0.77 (95% CI 0.72‐0.81; PI 0.57‐0.89), specificity 0.82 (95% CI 0.78‐0.86; PI 0.60‐0.93); DOR, 15.49 (95% CI 11.16‐21.50); and area under the curve (AUC), 0.86 (95% CI 0.83‐0.89). External sensitivity was 0.73 (95% CI 0.61‐0.81; PI 0.34‐0.93), specificity 0.83 (95% CI 0.73‐0.90; PI 0.36‐0.98), DOR 12.93 (95% CI 7.25‐23.07), and AUC 0.84 (95% CI 0.79‐0.89). DOR PIs were 5.40‐44.44 and 2.40‐69.62, respectively. Thirty-two studies were conducted in China, and 12 had high validation risk of bias. All GRADE-assessed outcomes had very low certainty. Selecting the first eligible imaging model reported in each article instead of the highest-AUC model showed modest decreases in average performance across 16 internal and 10 external datasets; incomplete alternative-model reporting limited interpretation. Single study algorithm estimates were reported descriptively, without ranking algorithms or interpreting their CIs as evidence of between study consistency or generalizability.

**Conclusions:**

By combining multiple neoadjuvant strategies and separating validation stages, this review extends earlier neoadjuvant chemoradiotherapy (NCRT)-focused syntheses and identifies priorities for further model evaluation. Imaging-based AI may support response assessment, but wide PIs, risk of bias, and very low evidence certainty preclude routine clinical adoption without prospective, geographically diverse validation. Future studies should evaluate prespecified models in independent populations, report clinically relevant decision thresholds, and assess calibration and patient outcomes. Until such evidence is available, these models should be considered investigational adjuncts to response assessment and should not independently guide organ preservation or omission of surgery after neoadjuvant treatment.

## Introduction

Esophageal cancer is one of the most lethal gastrointestinal malignancies worldwide, causing over 500,000 deaths annually, with an overall 5-year survival rate below 25% [1]. The main histological subtypes are esophageal squamous cell carcinoma (ESCC) and esophageal adenocarcinoma. Surgery remains the primary treatment, but for locally advanced cases, it is often insufficient for long-term survival. The European Society for Medical Oncology (ESMO) and the National Comprehensive Cancer Network (NCCN) recommend preoperative neoadjuvant therapy for patients with locally advanced esophageal cancer [2-4]. If patients achieve a pathological complete response (pCR) after neoadjuvant therapy, active surveillance or organ-preserving strategies may be considered [5]. However, pCR rates remain limited, ranging from 43%‐49% in squamous cell carcinoma and 23% in adenocarcinoma [6]. Early and accurate identification of pCR is critical to guide treatment decisions and promote precision clinical management.

pCR is defined as the absence of viable tumor cells in both the primary lesion and regional lymph nodes after neoadjuvant therapy. In clinical practice, treatment response is often evaluated using Response Evaluation Criteria in Solid Tumors (RECIST) version 1.1. However, RECIST 1.1 indicates that primary lesions in hollow organs are often difficult to measure accurately [7]. Consequently, esophageal cancer lesions are frequently classified as nonmeasurable. After neoadjuvant therapy, tumors may achieve pCR, yet residual soft-tissue densities are often still observed on computed tomography (CT) images, predominantly representing fibrosis or inflammatory changes rather than viable tumor. RECIST 1.1 relies on morphological changes, making it difficult to accurately distinguish cases that have achieved pCR, thereby limiting its predictive performance.

AI has been successfully applied in medicine, enabling automated recognition and analysis of complex medical data [8]. Radiomics combines AI and medical imaging to extract quantitative image features using algorithms, potentially reflecting underlying pathophysiology and revealing pathogenesis [9]. Recent machine learning (ML)-based radiomic analyses have demonstrated improved accuracy in predicting therapy response and assessing prognosis [10]. Imaging-based AI models show potential for predicting pCR in esophageal cancer following neoadjuvant therapy. For resectable locally advanced esophageal cancer, neoadjuvant chemoradiotherapy (NCRT) combined with surgery is the standard treatment [11]. Recent studies indicate that neoadjuvant immunotherapy combined with chemotherapy (NICT) also shows favorable efficacy and safety in locally advanced ESCC [12-16]. If patients achieve pCR, they may be considered for organ preservation or active surveillance [17]. However, differences in imaging modalities, AI algorithms, and treatment regimens result in fluctuating predictive accuracy of AI models, making their translation into clinically useful tools challenging.

Previous reviews have evaluated the performance of radiomics-based models for predicting pCR after NCRT [11,18]; however, these reviews were largely confined to this single treatment modality. With the rapid development of novel therapeutic strategies such as NICT and neoadjuvant chemotherapy (NACT), the predictive value of AI models across different treatment modalities remains to be systematically assessed. Furthermore, existing reviews have seldom distinguished between internal and external validation results, offering limited discussion on model generalizability. Thus, this meta-analysis aims to assess the predictive performance of imaging-based AI models for pCR in esophageal cancer after neoadjuvant therapy across multiple neoadjuvant treatment strategies for the first time, and to separately extract and synthesize results from internal and external validation cohorts to rigorously evaluate model generalizability, as well as factors affecting their performance.

## Methods

### Study Design

This meta-analysis was conducted in strict accordance with the PRISMA-DTA (Preferred Reporting Items for Systematic Reviews and Meta-Analyses for Diagnostic Test Accuracy) guidelines [19]. The PRISMA-DTA checklist is provided in Checklist 1. The PRISMA (Preferred Reporting Items for Systematic Reviews and Meta-Analyses) 2020 for Abstracts checklist is provided in Checklist 2. Search reporting followed the PRISMA-S (Preferred Reporting Items for Systematic Reviews and Meta-Analyses literature search extension) extension [20], and the completed PRISMA-S checklist is provided in Checklist 3. Additionally, the study protocol has been registered in the PROSPERO (International Prospective Register of Systematic Reviews) database (CRD420251207710). Deviations from the registered protocol comprised an update and expansion of the literature search during revision (final search: August 7, 2026) and the additional calculation of prediction intervals (PIs) for pooled sensitivity and specificity.

### Information Sources and Search Strategy

We conducted a systematic literature search across 4 databases: PubMed, Embase, Web of Science, and the Cochrane Library, with literature retrieval completed by 2 independent reviewers (ZL and XG) until August 7, 2026. Our search strategy comprised 3 groups of keywords: AI-related terms (eg, artificial intelligence, machine learning, and deep learning); disease-specific terms (eg, esophageal neoplasms, esophageal adenocarcinoma, and ESCC); and neoadjuvant therapy-related terms (eg, neoadjuvant therapy, NACT, neoadjuvant chemoimmunotherapy, and NCRT). A search strategy combining free-text keywords and MeSH was used to ensure accuracy, as detailed in Table S1 in Multimedia Appendix 1. Initial searches posed no restrictions on language or publication year. As an additional information source, citation searching was performed by screening the reference lists of the included studies and of similar published systematic reviews and meta-analyses on the same topic. No study registries or other online resources were searched, no search filters were used, and the search strategy did not undergo formal peer review.

### Eligibility Criteria

Inclusion criteria carefully adhered to the PITROS (participants, index test, target conditions, reference standard, outcomes, and settings) framework. Participants (P): The participants in this study are patients with esophageal cancer undergoing neoadjuvant therapies, which include NACT, neoadjuvant immunotherapy plus chemoradiotherapy (NICRT), NCRT, and neoadjuvant immunochemotherapy. Index test (I): This study uses AI techniques, specifically ML and deep learning (DL), to analyze imaging data (CT/magnetic resonance imaging [MRI]) to predict the pCR in patients with esophageal cancer. Target condition (T): The positive group is defined as patients with a pathologically confirmed pCR, while the negative group consists of patients who did not achieve a pCR. Reference standard (R): Pathological assessment was used as the reference standard to determine whether patients had achieved pCR. Outcomes (O): The primary outcomes include patient-based analysis of sensitivity, specificity, diagnostic odds ratio (DOR), and the area under the curve (AUC). Setting (S): The study setting includes retrospective or prospective data sources, covering public databases or local hospitals.

Exclusion criteria included studies with titles and abstracts that were clearly unrelated to the research topic, as well as specific types of literature (eg, reviews, case reports, conference abstracts, meta-analyses, and letters to editors). Non-English studies were excluded due to lack of accessibility. This English-only restriction was considered a potential limitation because it may have excluded relevant evidence. Furthermore, research related to tumors at the gastroesophageal junction and studies with overlapping samples were also excluded. The screening process was conducted independently by 2 reviewers (LW and JZ), who first assessed the relevance of titles and abstracts and then evaluated the full texts based on the inclusion and exclusion criteria. Discrepancies among reviewers were resolved through discussion, and if consensus could not be reached, a third reviewer (FR) determined the final decision.

### Risk of Bias and Certainty Assessment

The quality of the included studies was rigorously evaluated using the updated 2025 PROBAST+AI tool (Prediction model Risk of Bias Assessment Tool plus Artificial Intelligence) [21]. This tool provides a comprehensive evaluation of both model development and validation phases, with each phase covering 7 key domains including participants and data sources, predictors, outcome assessment, and analytical methods. Detailed evaluation procedures and results are shown in Tables S2 and S3 in Multimedia Appendix 1.

To ensure objectivity and accuracy, 2 reviewers (ZL and JZ) independently assessed the risk of bias in included studies using the PROBAST+AI tool. Discrepancies were resolved through discussion and consensus, thereby enhancing the reliability and consistency of the final assessments. We used the GRADE (Grading of Recommendations Assessment, Development, and Evaluation) tool to evaluate the certainty of sensitivity, specificity, and DOR, focusing on 5 key domains: risk of bias, indirectness, inconsistency, imprecision, and publication bias [22]. Downgrading in any domain resulted in reduced overall evidence certainty.

### Data Collection Process and Data Items

Two reviewers (XG and ZL) independently extracted data from all included studies to minimize bias and enhance the accuracy and reliability of the dataset. Any disagreements between the 2 reviewers were resolved through consultation with a third reviewer (LW), who served as an arbitrator to facilitate consensus. Extracted data included the first author, publication year, patient origin country, type of histopathology, study design, type of therapy, reference standard, No. of pCR patients, and analysis approach. Technical information was also recorded, including type of imaging, imaging technical details, data source, type of AI model, data splitting method, AI method, the best-performing AI algorithms, as well as the number of patients in the training, internal validation, and external validation sets, with corresponding counts of true positives (TPs), true negatives (TNs), false positives (FPs), and false negatives (FNs). For studies lacking data required for meta-analysis, we contacted corresponding authors via email to request the missing information.

### Index Test and Outcome Measures

The primary outcomes included sensitivity, specificity, AUC, and DOR for both internal and external validation sets. Internal validation referred to evaluation within the development data or a held-out subset, whereas external validation referred to evaluation in an independent cohort from a different institution or time period and therefore provided a more direct assessment of generalizability. Sensitivity (TP rate) was used to assess the ability of an AI model to correctly identify pCR cases and was calculated as TP/(TP+ FN). Specificity (TN rate) reflected the probability of correctly identifying non-pCR cases and was calculated as TN/(TN+ FP). The AUC served as a comprehensive measure of the model’s ability to distinguish between positive and negative cases. DOR, calculated as the ratio of the positive likelihood ratio (LR+) to the negative likelihood ratio (LR−), was used to evaluate diagnostic performance. For studies with multiple nonoverlapping patient datasets (eg, internal validation and external validation), each dataset was treated as an independent dataset and included in the analysis. Additionally, for studies evaluating multiple AI models, only the model with the highest AUC from the internal or external validation sets was extracted. Internal and external validation were synthesized separately. Validation sample counts were summed within each group; training samples and repeated cross-validation folds were not added again. The 2 validation groups contributed 2668 validation observations in total (1891+777). This sum was not interpreted as a verified count of unique patients because patient overlap across validation datasets and publications could not be fully assessed from the available study-level reports.

### Synthesis Methods and Statistical Analysis

A bivariate random-effects model was applied in the meta-analysis to assess the diagnostic performance of AI models for predicting pCR in patients with esophageal cancer. Because sensitivity and specificity were jointly modeled, the bivariate random-effects framework was retained as the primary model rather than splitting the outcomes into separate univariate analyses solely to apply the Hartung-Knapp-Sidik-Jonkman (HKSJ) correction; HKSJ is primarily designed for conventional univariate random-effects meta-analysis and is not a general replacement correction for a bivariate diagnostic-accuracy model. AUC was derived from the summary receiver operating characteristic (SROC) curve, and DOR was derived from the difference between the model coefficients for logit sensitivity and logit FP rate; conventional HKSJ was therefore not directly applicable to these derived outcomes. Predictive results were pooled for the internal and external validation cohorts. Forest plots were generated to visually present pooled sensitivity, specificity, and DOR, while an SROC curve displayed the summary point and joint confidence and prediction regions. Z-tests were used to compare pooled sensitivity, specificity, DOR, and AUC values, with statistical significance defined as *P*<.05. Inconsistency was assessed using outcome-specific *I*² values based on Cochran Q statistic. Q was calculated for logit sensitivity, logit specificity, and log DOR. For sensitivity and specificity, 0.5 was added to both event and nonevent counts if either count was zero. For DOR, 0.5 was added to all 4 cells if any cell was zero. *I*² estimated the proportion of observed variability attributable to between-study differences rather than sampling error, not the absolute magnitude of heterogeneity. Between-study variance (τ²), its square root (τ), and PIs were obtained from the bivariate model to describe variation on the corresponding effect scales [23]. Meta-regression analyses considered AI method (ML vs DL), AI model (radiomic and clinical models vs radiomic-only models), and contrast-enhanced imaging (yes vs no), using the categories reported in the supplementary meta-regression tables. Bubble plots were generated to assess temporal trends in AI model AUCs, and radar charts were constructed to depict the distribution of ML and DL algorithms. The clinical utility of the AI model was evaluated using a Fagan nomogram, with a pretest probability of 34%, close to the median pCR rate across 43 internal and external validation datasets (34.8%; IQR 27.7%-40.6%). Although PIs were not specified in our original PROSPERO registration protocol, we additionally calculated marginal 95% PIs for sensitivity, specificity, and DOR in both validation groups to better quantify the distribution of effects across different populations and settings, as recommended by recent methodological guidance on the reporting of PIs [24,25]. Potential small-study effects and funnel-plot asymmetry were assessed using Deeks test; asymmetry was not interpreted as evidence of publication bias alone [26,27]. The primary bivariate estimates, PIs, and model-selection sensitivity analysis were obtained using R (R Foundation for Statistical Computing; version 4.5.2). AUC CIs for the primary analyses were estimated using the delta method. Secondary analyses used Stata (StataCorp LLC; versions 15.1 and 17.0) with the Midas and Metadta modules. Data visualization used the *ggplot2* and *tidyverse* packages in R.

Sensitivity analyses excluded outliers identified in bivariate box plots, excluded low-quality studies, and included only ESCC cohorts (Table 1). We also compared the highest-AUC model with the first eligible imaging model reported in each article, using the same validation cohorts and pCR definitions (Tables S9 and S10 in Multimedia Appendix 1). Models were selected in table order, or in Results order when no eligible performance table was available; comparisons were limited to datasets with usable counts for both selections.

**Table 1.** Sensitivity analyses of pooled diagnostic performance for pCR^a^ in internal and external validation cohorts, including analyses excluding outliers or low-quality studies and analyses restricted to ESCC^b^ studies.

Sensitivity analysis	Datasets, n	Sensitivity (95% CI)	Specificity (95% CI)	AUC^c^ (95% CI)	DOR^d^ (95% CI)
Internal validation sets					
Overall	27	0.77 (0.72‐0.81)	0.82 (0.78‐0.86)	0.86 (0.83‐0.89)	15.49 (11.16‐21.50)
Without outliers in Bivariate box plot^e^	21	0.77 (0.72‐0.82)	0.82 (0.78‐0.86)	0.86 (0.83‐0.89)	15.98 (11.53‐22.14)
Without low-quality studies^f^	17	0.76 (0.70‐0.81)	0.83 (0.77‐0.87)	0.86 (0.83‐0.89)	15.15 (10.04‐22.88)
Including only ESCC patients^g^	24	0.76 (0.72‐0.80)	0.83 (0.79‐0.87)	0.85 (0.82‐0.88)	16.06 (11.26‐22.90)
External validation sets					
Overall	16	0.73 (0.61‐0.81)	0.83 (0.73‐0.90)	0.84 (0.79‐0.89)	12.93 (7.25‐23.07)
Without outliers in Bivariate box plot^e^	13	0.71 (0.57‐0.82)	0.81 (0.75‐0.87)	0.84 (0.80‐0.88)	10.72 (6.63‐17.33)
Without low-quality studies^f^	12	0.72 (0.58‐0.83)	0.82 (0.69‐0.90)	0.84 (0.80‐0.87)	11.85 (6.17‐22.79)
Including only ESCC patients^g^	14	0.74 (0.62‐0.83)	0.83 (0.71‐0.90)	0.85 (0.81‐0.88)	13.76 (7.10‐26.66)

a pCR: pathological complete response.

b ESCC: esophageal squamous cell carcinoma.

c AUC: area under the curve.

d DOR: diagnostic odds ratio.

e The following studies were excluded for internal validation sets: Wang et al, 2024 [28], Qi et al, 2025 [29], Qi et al, 2024 [30], Fan et al, 2024 [31], Liu et al, 2025 [32], and Qi et al, 2025 [33]. Also, the following studies were excluded for external validation sets: Zhang et al, 2025 [34], Ling et al, 2025 [35], and Zhao et al, 2025 [36].

f Low quality means that the study was rated as having at least one high-risk item in the PROBAST+AI (Prediction Model Risk of Bias Assessment Tool plus Artificial Intelligence) tool.

g The following studies were excluded for internal validation sets: Qi et al, 2025 [29], Li et al, 2024 [37], and﻿ Yue et al, 2022 [38]. Also, the following studies were excluded for external validation sets: Kong et al, 2024 [39] and Li et al, 2024 [37].

## Results

### Study Selection

The search strategy yielded 1680 records, including 411 from PubMed, 734 from Embase, 521 from Web of Science, and 14 from the Cochrane Library. After removing 693 duplicates using EndNote 20 (Clarivate), 987 records were screened by title and abstract, and 919 were excluded because they were case reports, letters, reviews, animal experiments, meta-analyses, or clearly irrelevant. Following a detailed full-text review of 68 articles, 35 studies were further excluded for nonimaging-based AI model research (n=8), inaccurate definition of pCR (n=12), gastroesophageal junction cancer with unclear pathology (n=7), unavailable diagnostic data (TP, FP, TN, FN; n=4), or overlapping samples (n=4). In addition, citation searching of the reference lists of similar published meta-analyses (ie, the studies included in those reviews) identified 113 additional records; after screening, 104 were removed as irrelevant, and the remaining 9 full-text articles were excluded because of sample overlap. Ultimately, 33 articles were included in this systematic review [16,28-59]. The literature screening process was systematically documented using a standardized PRISMA flow diagram, as shown in Figure 1. We identified 17 non-English or mixed-language reports; none met the other eligibility criteria.

**Figure 1.**
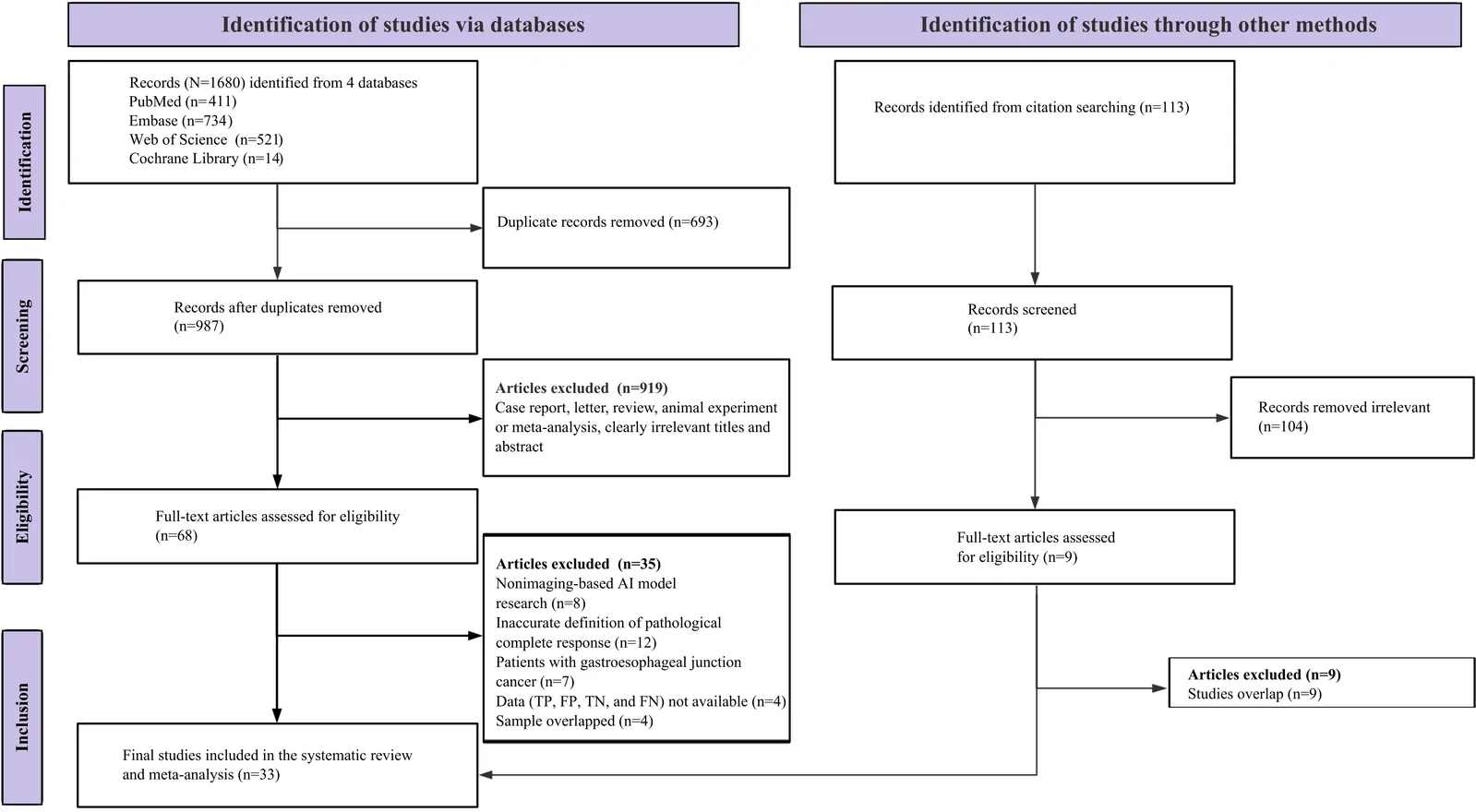
PRISMA (Preferred Reporting Items for Systematic Reviews and Meta-Analyses) flow diagram illustrating the identification, screening, eligibility assessment, and inclusion of studies evaluating imaging-based AI for pathological complete response in esophageal cancer after neoadjuvant therapy. FN: false negative; FP: false positive; TN: true negative; TP: true positive.

### Study Characteristics

A total of 33 eligible studies [16,28-59] were included. Internal validation was reported in 27 studies comprising 27 datasets and 1891 patients [16,28-34,36-38,40,41,43,44,46,48,49,51-59], while external validation was reported in 16 studies with 16 datasets involving 777 patients [16,33-37,39,41-43,45,47,50,53,55,57]. Nearly all studies were conducted in China (32/33) [16,28-53,55-59], with one study [54] from Japan, and studies were published between 2019 and 2026. Most included studies were retrospective, using pathological results as the reference standard. CT was the most commonly used imaging modality (30/33, 91%), most commonly with contrast enhancement (28/33, 85%). In both internal and external validation sets, ML was the predominant approach for building AI models. Logistic regression (LR) was the most frequently used algorithm in internal validation sets (9/27, 33%), whereas Extreme Gradient Boosting (XGBoost) was the most frequent in external validation sets (4/16, 25%). A detailed summary of the study, patient, and technical characteristics is presented in Table 2 and Tables S4-S6 in Multimedia Appendix 1. Ten publications contributed to both validation groups, yielding 43 validation datasets across 33 publications. The 2 validation counts summed to 2668 observations (1891+777); the number of unique patients across datasets and publications could not be verified.

**Table 2.** Characteristics of the included studies evaluating imaging-based AI for pCR^a^ in patients with esophageal cancer after neoadjuvant therapy, including study design, histology, treatment, validation cohort, and patient distribution.

Author and year	Country	Study design	Type of histopathology	Type of therapy	Reference standard	Analysis	Training^b^	Internal validation^b^	External validation^b^	Number of pCR patients
Wang et al, 2023 [40]	China	Retro^c^	ESCC^d^	NCRT^e^	Pathology	PB^f^	112	112	NA^g^	Training: 48 Internal validation: 48
Ou et al, 2024 [41]	China	Retro	ESCC	NACT^h^	Pathology	PB	188	83	55	Training: 65 Internal validation: 29 External validation: 21
Wang et al, 2024 [28]	China	Retro	ESCC	NICRT^i^	Pathology	PB	60	60	NA	Training: 28 Internal validation: 28
Zhang et al, 2025 [34]	China	Retro	ESCC	NICT^j^	Pathology	PB	469	118	34	Training: 105 Internal validation: 18 External validation: 11
Qi et al, 2025 [29]	China	Retro	EC^k^	NICT	Pathology	PB	160	54	NA	Training: 45 Internal validation: 15
Zheng et al, 2024 [42]	China	Retro	ESCC	NICT	Pathology	PB	93	NA	47	Training: 27 External validation: 13
Zhang et al, 2024 [43]	China	Retro	ESCC	NCRT	Pathology	PB	111	46	33	Training: 50 Internal validation: 16 External validation: 12
Qi et al, 2024 [30]	China	Retro	ESCC	NCRT	Pathology	PB	100	26	NA	Training: 53 Internal validation: 13
Lu et al, 2024 [44]	China	Retro	ESCC	NICT	Pathology	PB	74	31	NA	Training: 24 Internal validation: 11
Liu et al, 2024 [45]	China	Retro	ESCC	NCRT	Pathology	PB	82	NA	73	Training: 39 External validation: 24
Liu et al, 2024 [46]	China	Retro	ESCC	NCRT	Pathology	PB	57	25	NA	Training: 27 Internal validation: 12
Kong et al, 2024 [39]	China	Retro	EC	NICT	Pathology	PB	85	NA	27	Training: 18 External validation: 6
Fan et al, 2024 [31]	China	Retro	ESCC	NCRT	Pathology	PB	186	46	NA	Training: 59 Internal validation: 17
Li et al, 2024 [37]	China	Retro	EC	NCRT	Pathology	PB	139	62	30	Training: 39 Internal validation: 14 External validation: 12
Li et al, 2023 [47]	China	Retro	ESCC	NACT	Pathology	PB	116	NA	27	Training: 59 External validation: 4
Li et al, 2023 [48]	China	Retro	ESCC	NICT	Pathology	PB	66	29	NA	Training: 27 Internal validation: 12
Yue et al, 2022 [38]	China	Retro	EC	NCRT	Pathology	PB	124	124	NA	Training: 49 Internal validation: 49
Li et al, 2021 [49]	China	Retro	ESCC	NCRT	Pathology	PB	121	121	NA	Training: 51 Internal validation: 51
Hu et al, 2020 [50]	China	Retro	ESCC	NCRT	Pathology	PB	161	NA	70	Training: 74 External validation: 31
Yang et al, 2019 [51]	China	Retro	ESCC	NCRT	Pathology	PB	44	11	NA	Training: 19 Internal validation: 4
Zhu et al, 2025 [16]	China	Retro	ESCC	NICT	Pathology	PB	161	69	41	Training: 46 Internal validation: 20 External validation: 12
Sun et al, 2024 [52]	China	NA	ESCC	NCRT	Pathology	PB	136	34	NA	Training: NA Internal validation: 14
Gu et al, 2025 [53]	China	Retro	ESCC	NICT	Pathology	PB	253	253	40	Training: 40 Internal validation: 40 External validation: 20
Hirata et al, 2025 [54]	Japan	Retro	ESCC	NCRT	Pathology	PB	70	70	NA	Training: 15 Internal validation: 15
Ling et al, 2025 [35]	China	Retro and Pro^l^	ESCC	NICT	Pathology	PB	110	NA	47	Training: 32 External validation: 8
Liu et al, 2025 [32]	China	Retro	ESCC	NICT	Pathology	PB	66	66	NA	Training: 18 Internal validation: 18
Liu et al, 2025 [55]	China	Retro	ESCC	NCRT	Pathology	PB	194	49	58	Training: 71 Internal validation: 18 External validation: 19
Qi et al, 2025 [33]	China	Retro	ESCC	NICT	Pathology	PB	181	115	39	Training: 43 Internal validation: 21 External validation: 13
Shi et al, 2026 [56]	China	Retro	ESCC	NCRT and NICRT	Pathology	PB	142	94	NA	Training: 63 Internal validation: 44
Xu et al, 2026 [57]	China	Retro	ESCC	NICT	Pathology	PB	107	47	81	Training: 24 Internal validation: 22 External validation: 19
Zhao et al, 2026 [58]	China	Retro	ESCC	NICT	Pathology	PB	81	41	NA	Training: 30 Internal validation: 15
Zhao et al, 2025 [36]	China	Retro	ESCC	NICT	Pathology	PB	171	40	75	Training: 56 Internal validation: 12 External validation: 22
Xu et al, 2025 [59]	China	Retro	ESCC	NICT	Pathology	PB	152	65	NA	Training: 44 Internal validation: 20

a pCR: pathological complete response.

b Number of total patients.

c Retro: retrospective.

d ESCC: esophageal squamous cell carcinoma.

e NCRT: neoadjuvant chemoradiotherapy.

f PB: patient-based.

g NA: not available.

h NACT: neoadjuvant chemotherapy.

i NICRT: neoadjuvant immunotherapy plus chemoradiotherapy.

j NICT: neoadjuvant immunotherapy combined with chemotherapy.

k EC: esophageal cancer.

l Pro: prospective.

### Risk of Bias and Certainty of Evidence

The PROBAST+AI quality evaluation tool was applied to assess the risk of bias, with results presented in Figure 2 and Tables S2 and S3 in Multimedia Appendix 1. For model development, 3.0% (1/33) [51] of studies were rated as high risk in the overall judgment of quality, and 3.0% (1/33) [54] showed high concern regarding applicability. For model validation, 36.4% (12/33) [30,31,35,39,42-44,46,48,51,52,58] of studies were assessed as high risk in the overall risk of bias judgment, and 3.0% (1/33) [54] demonstrated high concern regarding applicability. Overall, high-risk items were relatively infrequent, with low-risk items predominating, indicating that the quality of the included literature was generally acceptable.

**Figure 2.**
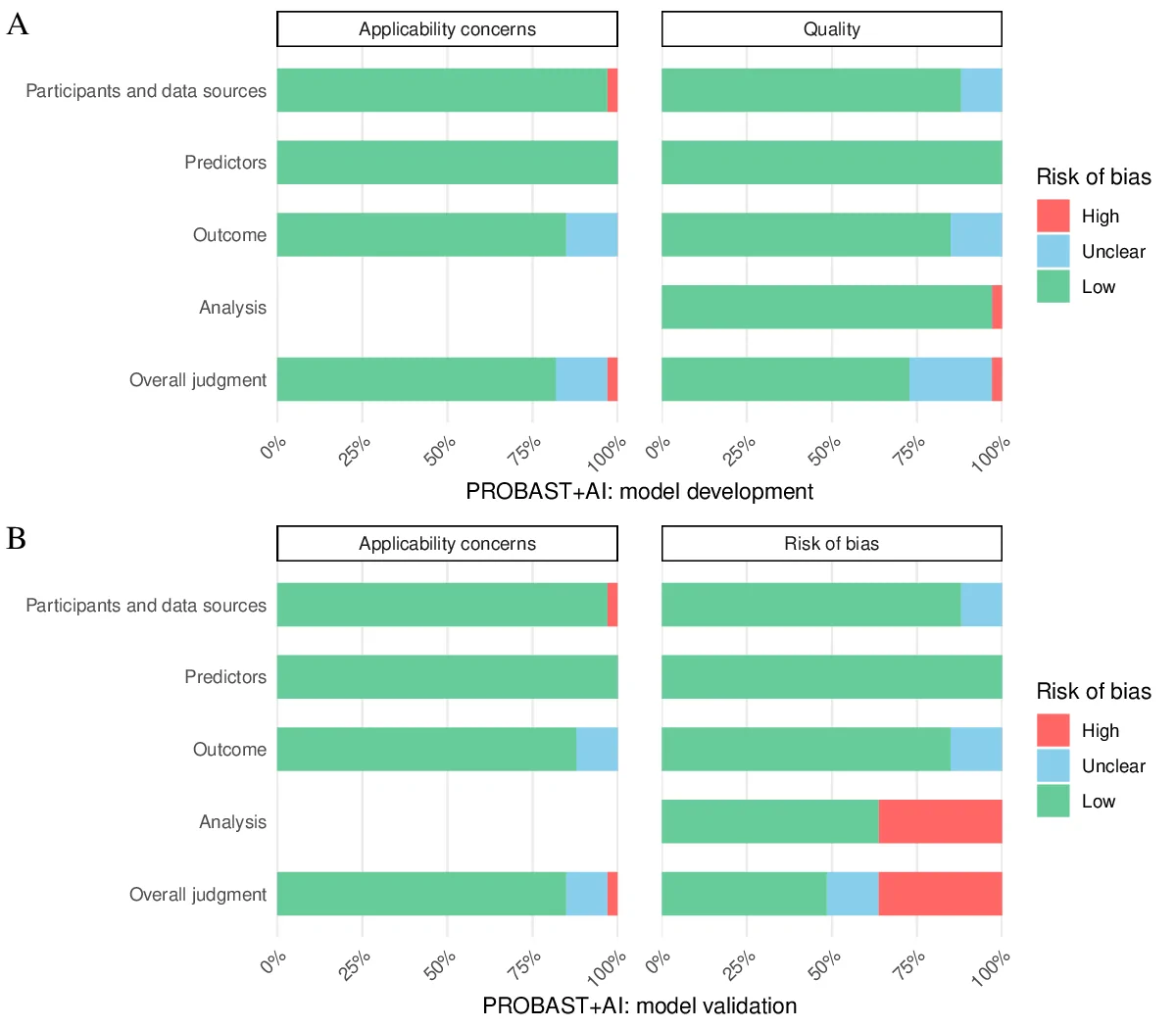
Risk of bias and applicability concerns assessment based on the PROBAST+AI tool for studies predicting pathological complete response in esophageal cancer. (A) Model development and (B) model validation. The green, blue, and red bars represent low risk, unclear risk, and high risk of bias or applicability concerns, respectively. PROBAST+AI: Prediction model Risk of Bias Assessment Tool plus Artificial Intelligence.

Certainty of evidence, assessed using GRADE criteria, was very low for all GRADE-assessed outcomes (Table 3). The main reasons for downgrading were a high risk of bias in multiple studies, unexplained inconsistency, indirectness, and imprecision of the CIs.

**Table 3.** GRADE^a^ certainty assessments for sensitivity, specificity, and DOR^b^^,^^c^.

Dataset and outcome	Risk of bias^d^	Inconsistency^e^	Indirectness^f^	Imprecision^g^	Publication bias^h^	Total downgrade	Final rating
Image-based AI for internal validation sets							
Sensitivity	1	0	1	1	0	3	Very low
Specificity	1	0	1	1	0	3	Very low
DOR	1	1	1	0	0	3	Very low
Image-based AI for external validation sets							
Sensitivity	1	1	0	1	0	3	Very low
Specificity	1	1	0	1	0	3	Very low
DOR	1	1	0	1	0	3	Very low

a GRADE: Grading of Recommendations Assessment, Development, and Evaluation.

b DOR: diagnostic odds ratio.

c Values indicate the number of levels downgraded; 0 indicates no downgrade, and 1 indicates one level.

d Risk of bias: assessed using tools such as PROBAST+AI to evaluate study design and methodology. If at least 1 study has a high risk of bias, the evidence level is downgraded by 1 level.

e Inconsistency: Between-study variation must be considered when interpreting summary estimates. PIs describe its practical implications; meta-regression associations alone do not establish a causal explanation. *I*² describes relative inconsistency and should not be interpreted as an absolute measure of heterogeneity.

f Indirectness evaluates whether study populations, interventions, or outcomes differ from the target research question. Downgrade if inconsistent outcome measures are used, for example, when sensitivity or specificity is indirectly estimated from the Youden Index derived from the receiver operating characteristic (ROC) curve.

g Imprecision: assessed based on CI width and sample size adequacy: For sensitivity/specificity: downgrade if the 95% CI width>0.20 or if CIs cross clinical thresholds (eg. 0.80). For diagnostic odds ratio (DOR): downgrade if the CI range exceeds one times the lower limit or includes values below 10.

h Publication bias: evaluated using Deeks funnel plot or similar tools. Downgrade by one level if the funnel plot shows significant asymmetry or if the *P* value in Deeks test is <.05.

### Predictive Performance of Different AI Models in Internal and External Validation Sets

In the internal validation sets, LR was the most frequently used AI algorithm (9/27, 33%), whereas XGBoost was the most frequent in the external validation sets (4/16, 25%; Figures 3A and 3B). Across internal validation sets, AUC values showed a slight increase from 2019 to 2026 (Figure 3C). By contrast, AUC values in external validation sets showed a downward trend over the same period, indicating that the generalizability of AI models remains a key concern (Figure 3D).

Comparisons of pooled diagnostic performance are shown in Figure 4. For internal validation, the pooled sensitivity of AI models was 0.77 (95% CI 0.72- 0.81; very low certainty), and the pooled specificity was 0.82 (95% CI 0.78-0.86; very low certainty; Figure S1A in Multimedia Appendix 1 and Table 3). The 95% PIs were 0.57-0.89 for sensitivity and 0.60-0.93 for specificity (Figure S1A in Multimedia Appendix 1). The pooled DOR was 15.49 (95% CI 11.16-21.50; very low certainty; Figure 5A and Table 3), and the pooled AUC was 0.86 (95% CI 0.83-0.89; Figure S2A in Multimedia Appendix 1). The DOR 95% PI was 5.40-44.44; the pooled estimate described average performance rather than a universal clinical value. Descriptive *I*^2^ values were 37.88%, 66.10%, and 34.19% for sensitivity, specificity, and DOR, respectively.

In the external validation sets, the pooled sensitivity was 0.73 (95% CI 0.61-0.81; very low certainty), and the pooled specificity was 0.83 (95% CI 0.73-0.90; very low certainty; Figure S1B in Multimedia Appendix 1 and Table 3). The 95% PIs were 0.34-0.93 for sensitivity and 0.36-0.98 for specificity (Figure S1B in Multimedia Appendix 1). The pooled DOR was 12.93 (95% CI 7.25-23.07; very low certainty; Figure 5B and Table 3), and the pooled AUC was 0.84 (95% CI 0.79-0.89; Figure S2B in Multimedia Appendix 1). The DOR 95% PI was 2.40-69.62. Descriptive *I*^2^ values were 51.78%, 78.38%, and 42.28% for sensitivity, specificity, and DOR, respectively.

**Figure 3.**
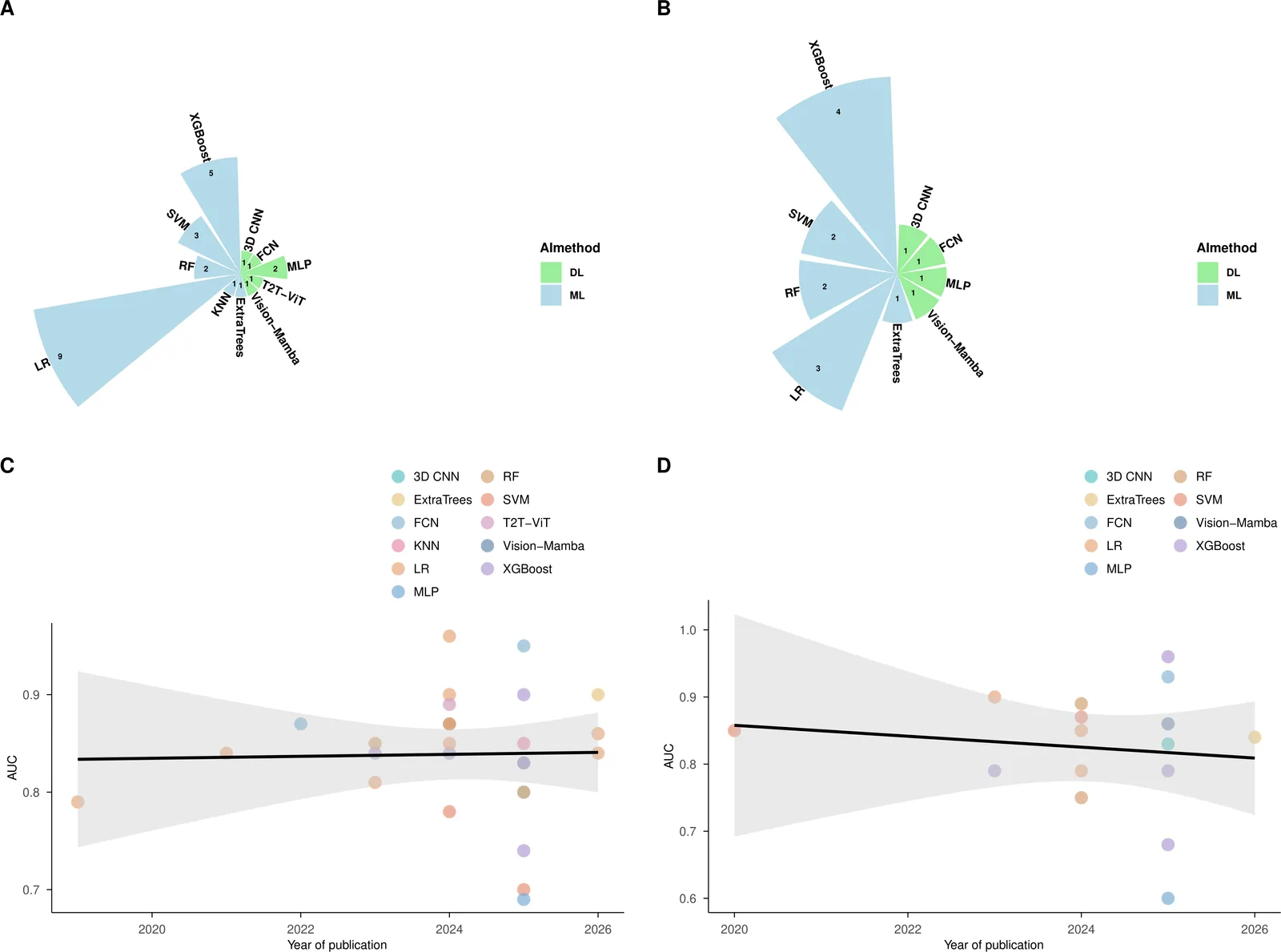
Distribution of AI algorithms and temporal trends in predictive performance. (A) Radar chart illustrating the distribution of AI algorithms in the internal validation set; (B) radar chart illustrating the distribution of AI algorithms in the external validation set; (C) bubble plot showing area under the curve (AUC) values over time in the internal validation set; and (D) bubble plot showing AUC values over time in the external validation set. The black line indicates the linear regression trend, with the shaded area denoting the 95% CI. 3D CNN: 3D convolutional neural network; AUC: area under the curve; DL: deep learning; KNN: K-nearest neighbors; FCN: fully convolutional network; LR: logistic regression; ML: machine learning; MLP: multilayer perceptron; RF: random forest; SVM: support vector machine; T2T-ViT: token-to-token Vision Transformer; XGBoost: Extreme Gradient Boosting.

**Figure 4.**
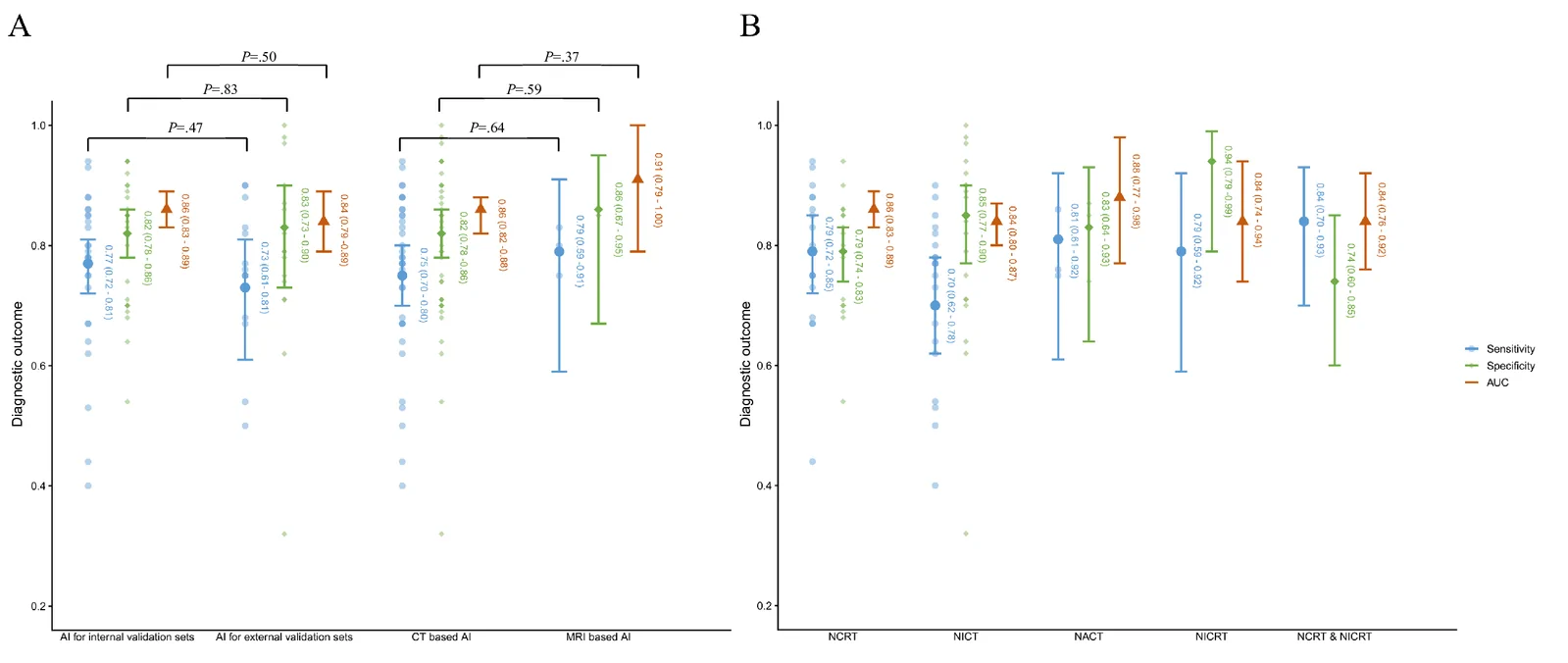
Plots comparing diagnostic performance for predicting pathological complete response across validation sets, imaging modalities, and neoadjuvant therapy regimens. (A) Sensitivity, specificity, and area under the curve (AUC) values for internal and external validation sets, as well as CT-based and MRI-based AI models, with *P* values indicating statistical comparisons between groups and (B) sensitivity, specificity, and AUC values categorized by different neoadjuvant therapy regimens. Individual points represent data from single studies, while the central markers and vertical error bars denote the pooled estimates and 95% CIs. AUC: area under the curve; CT: computed tomography; MRI: magnetic resonance imaging; NACT: neoadjuvant chemotherapy; NCRT: neoadjuvant chemoradiotherapy; NICT: neoadjuvant immunochemotherapy; NICRT: neoadjuvant immunochemoradiotherapy; pCR: pathological complete response.

**Figure 5.**
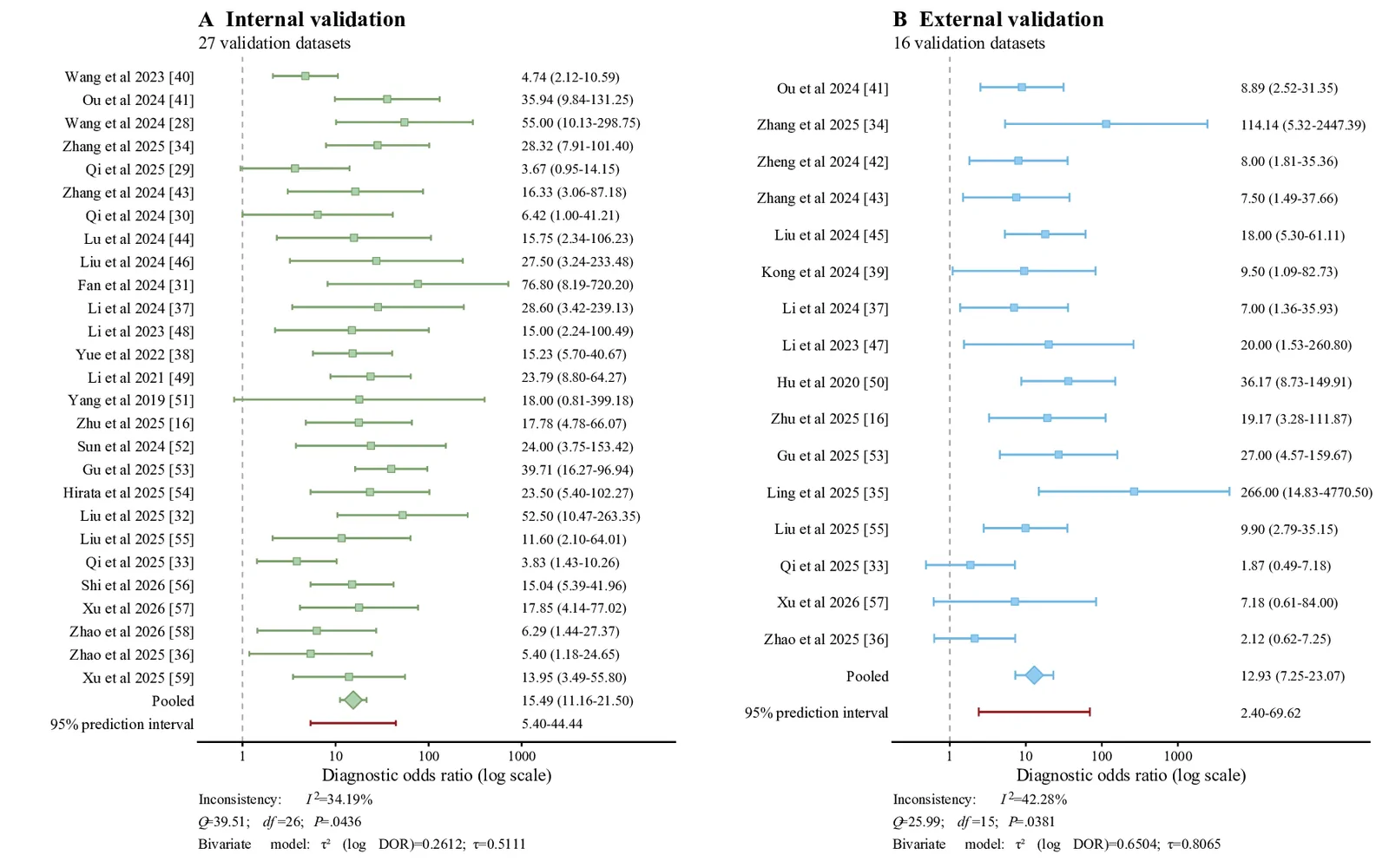
Diagnostic odds ratio forest plots for internal (A) and external (B) validation [16,28-59]. Squares and horizontal bars show individual-study estimates and 95% CIs. Diamonds show the pooled diagnostic odds ratio (DOR) and its 95% CI. The separate red line below the pooled estimate shows the 95% PI for a new study with similar conditions. The DOR and both intervals were derived from the same bivariate model, including the covariance between sensitivity and false-positive rate. Outcome-specific *I*² describes inconsistency on the log DOR scale, while *Q,* its degrees of freedom, and its *P* value are reported alongside it. The between-study variance (*τ*²) and standard deviation (τ) were derived from the bivariate model. DOR: diagnostic odds ratio; PI: prediction interval.

Statistical comparison revealed no significant differences between internal and external validation sets in sensitivity (*P*=.47), specificity (*P*=.83), DOR (*P*=.60), or AUC (*P*=.50; Figure 4A). Statistical significance should not be equated with clinically meaningful improvement; clinical relevance also depends on heterogeneity, evidence certainty, calibration, decision thresholds, and clinical consequences.

### Subgroup Analyses

Algorithm-specific estimates are shown in Table 4 and Figure S3 in Multimedia Appendix 1. Results from a single dataset are explicitly identified as single-study estimates, with CIs reflecting within-study sampling uncertainty; they are not pooled estimates. The small number of datasets in several subgroups precluded reliable ranking of algorithms. For different imaging modalities, CT-based models achieved a pooled sensitivity of 0.75 (95% CI 0.70‐0.80), specificity of 0.82 (95% CI 0.78‐0.86), and AUC of 0.86 (95% CI 0.82‐0.88). MRI-based models showed a pooled sensitivity of 0.79 (95% CI 0.59‐0.91), specificity of 0.86 (95% CI 0.67‐0.95), and AUC of 0.91 (95% CI 0.79‐1.00). No statistically significant differences were observed between the 2 modalities for sensitivity (*P*=.64), specificity (*P*=.59), or AUC (*P*=.37; Figure 4A). NACT had the numerically highest pooled AUC (0.88, 95% CI 0.77‐0.98) among the treatment subgroups (Figure 4B).

**Table 4.** Subgroup diagnostic performance of imaging-based AI algorithms for pCR^a^ in internal and external validation cohorts of patients with esophageal cancer after neoadjuvant therapy.

AI algorithms	Studies, n	Sensitivity (95% CI)	Specificity (95% CI)
Internal validation sets			
LR^b^	9	0.76 (0.68‐0.82)	0.80 (0.74‐0.85)
XGBoost^c^	5	0.77 (0.67‐0.85)	0.81 (0.74‐0.87)
SVM^d^	3	0.73 (0.59‐0.84)	0.80 (0.69‐0.88)
RF^e^	2	0.81 (0.60‐0.92)	0.76 (0.60‐0.87)
MLP^f^	2	0.85 (0.72‐0.93)	0.67 (0.53‐0.79)
Vision-Mamba	1	0.78 (0.52‐0.94)^g^	0.89 (0.81‐0.94)^g^
T2T-ViT^h^	1	0.86 (0.57‐0.98)^g^	0.80 (0.56‐0.94)^g^
FCN^i^	1	0.77 (0.62‐0.89)^g^	0.92 (0.88‐0.95)^g^
KNN^j^	1	0.80 (0.52‐0.96)^g^	0.85 (0.73‐0.94)^g^
3D CNN^k^	1	0.44 (0.22‐0.69)^g^	0.94 (0.79‐0.99)^g^
ExtraTrees^l^	1	0.77 (0.55‐0.92)^g^	0.84 (0.64‐0.95)^g^
External validation sets			
LR	3	0.70 (0.49‐0.86)	0.82 (0.69‐0.90)
XGBoost	4	0.74 (0.59‐0.85)	0.80 (0.70‐0.87)
SVM	2	0.85 (0.71‐0.92)	0.77 (0.63‐0.87)
RF	2	0.72 (0.55‐0.85)	0.83 (0.70‐0.92)
MLP	1	0.82 (0.60‐0.95)^g^	0.32 (0.20‐0.46)^g^
Vision-Mamba	1	0.73 (0.39‐0.94)^g^	1.00 (0.85‐1.00)^g^
T2T-ViT	0	—^m^	—
FCN	1	0.90 (0.68‐0.99)^g^	0.75 (0.51‐0.91)^g^
KNN	0	—	—
3D CNN	1	0.68 (0.43‐0.87)^g^	0.82 (0.66‐0.92)^g^
ExtraTrees	1	0.11 (0.01‐0.33)^g^	0.98 (0.91‐1.00)^g^

a pCR: pathological complete response.

b LR: logistic regression.

c XGBoost: extreme gradient boosting.

d SVM: support vector machine.

e RF: random forest.

f MLP: multilayer perceptron.

g These are single-study estimates, not pooled estimates; their CIs reflect within-study sampling uncertainty. Estimates from the remaining small subgroups are exploratory and should not be used to rank algorithms.

h T2T-ViT: token-to-token Vision Transformer.

i FCN: fully convolutional network.

j KNN: K-nearest neighbors.

k 3D CNN: 3D convolutional neural network.

l ExtraTrees: extremely randomized trees.

m N/A: not available.

### Heterogeneity and Sources of Variation

Meta-regression analyses indicated that, in the internal validation sets, performance was associated with AI method (ML, 21 datasets, vs DL, 6 datasets; sensitivity *P*=.01, specificity *P*<.001) and AI model (radiomic and clinical models, 8 datasets, vs radiomic-only models, 19 datasets; *P*<.001 for both), whereas the use of contrast-enhanced imaging was not statistically significant (*P*=.13 and *P*=.11; Table S7 in Multimedia Appendix 1). Bivariate box plots further identified the studies by Wang et al [28], Qi et al [29], Qi et al [30], Fan et al [31], Liu et al [32], and Qi et al [33] as potential contributors to the observed heterogeneity (Figure 6A).

**Figure 6.**
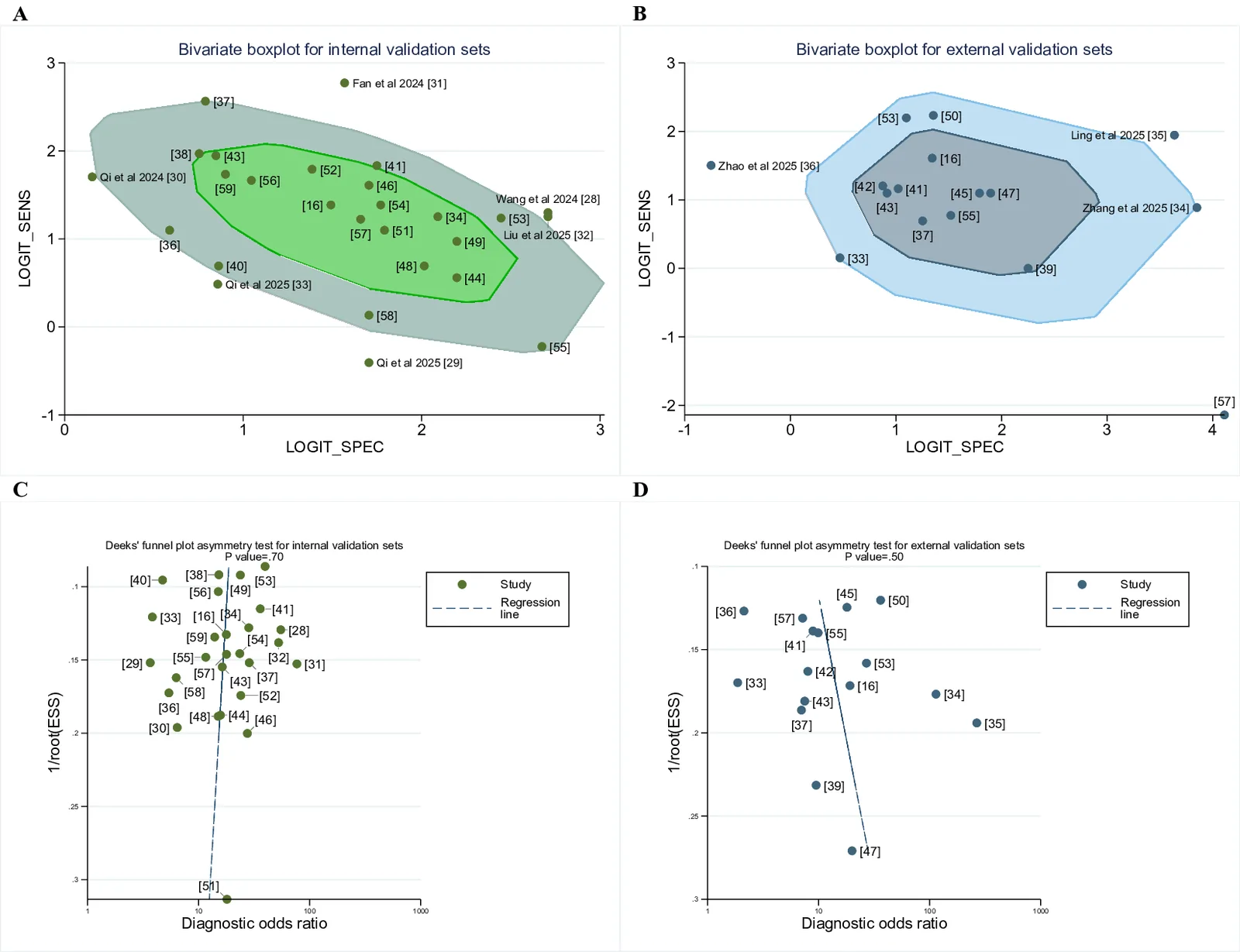
Bivariate boxplots and Deeks funnel plots for evaluating heterogeneity and small-study effects and possible publication bias in the internal and external validation sets [16,28-59]. (A) Bivariate boxplot for the internal validation sets; (B) bivariate boxplot for the external validation sets; (C) Deeks funnel plot for the internal validation sets; and (D) Deeks funnel plot for the external validation sets. Regression lines and associated *P* values in the funnel plots indicate funnel-plot asymmetry; these results may reflect small-study effects and should not be interpreted as proof of publication bias alone.

In the external validation sets, the meta-regression did not identify statistically significant sources of heterogeneity (ML, 12 datasets, vs DL, 4 datasets: *P*=.13 and *P*=.97; radiomic and clinical models, 3 datasets, vs radiomic-only models, 13 datasets: *P*=.81 and *P*=.37; contrast-enhanced imaging: *P*=.83 and *P*>.99; Table S8 in Multimedia Appendix 1). Bivariate box plots suggested that Zhang et al [34], Ling et al [35], and Zhao et al [36] may represent potential sources of heterogeneity (Figure 6B).

### Sensitivity Analyses

Table 1 and Figure S4-S6 in Multimedia Appendix 1 summarize the results of the sensitivity analyses. These analyses were performed separately by excluding low-quality studies, removing outliers identified in bivariate box plots, and including only studies on ESCC. In the internal validation sets, sensitivity ranged from 0.76 to 0.77, specificity from 0.82 to 0.83, AUC from 0.85 to 0.86, and DOR from 15.15 to 16.06 across the sensitivity analyses, indicating that the results were stable. In the external validation sets, sensitivity ranged from 0.71 to 0.74, specificity from 0.81 to 0.83, AUC from 0.84 to 0.85, and DOR from 10.72 to 13.76, suggesting that the results were consistent.

Thirty of 33 [16,28-40,42-48,50-53,55-59] publications reported multiple eligible imaging models. Comparing the highest-AUC model with the first eligible imaging model reported in each article was possible for 16 internal and 10 external datasets; the selected model changed in 4 and 3 datasets, respectively. Average performance decreased modestly with the alternative selection (Tables S9 and S10 in Multimedia Appendix 1).

### Clinical Utility and Small-Study Effects

Deeks funnel plot test showed no evidence of funnel-plot asymmetry suggesting small-study effects in either the internal validation sets (*P*=.70) or the external validation sets (*P*=.50; Figures 6C and 6D). Based on a prior probability of 34%, the Fagan nomogram for the internal validation sets showed a positive post probability of 69% and a negative post probability of 13% (Figure 7A). For the external validation sets, the Fagan nomogram indicated a positive post probability of 69% and a negative post probability of 15% (Figure 7B).

**Figure 7.**
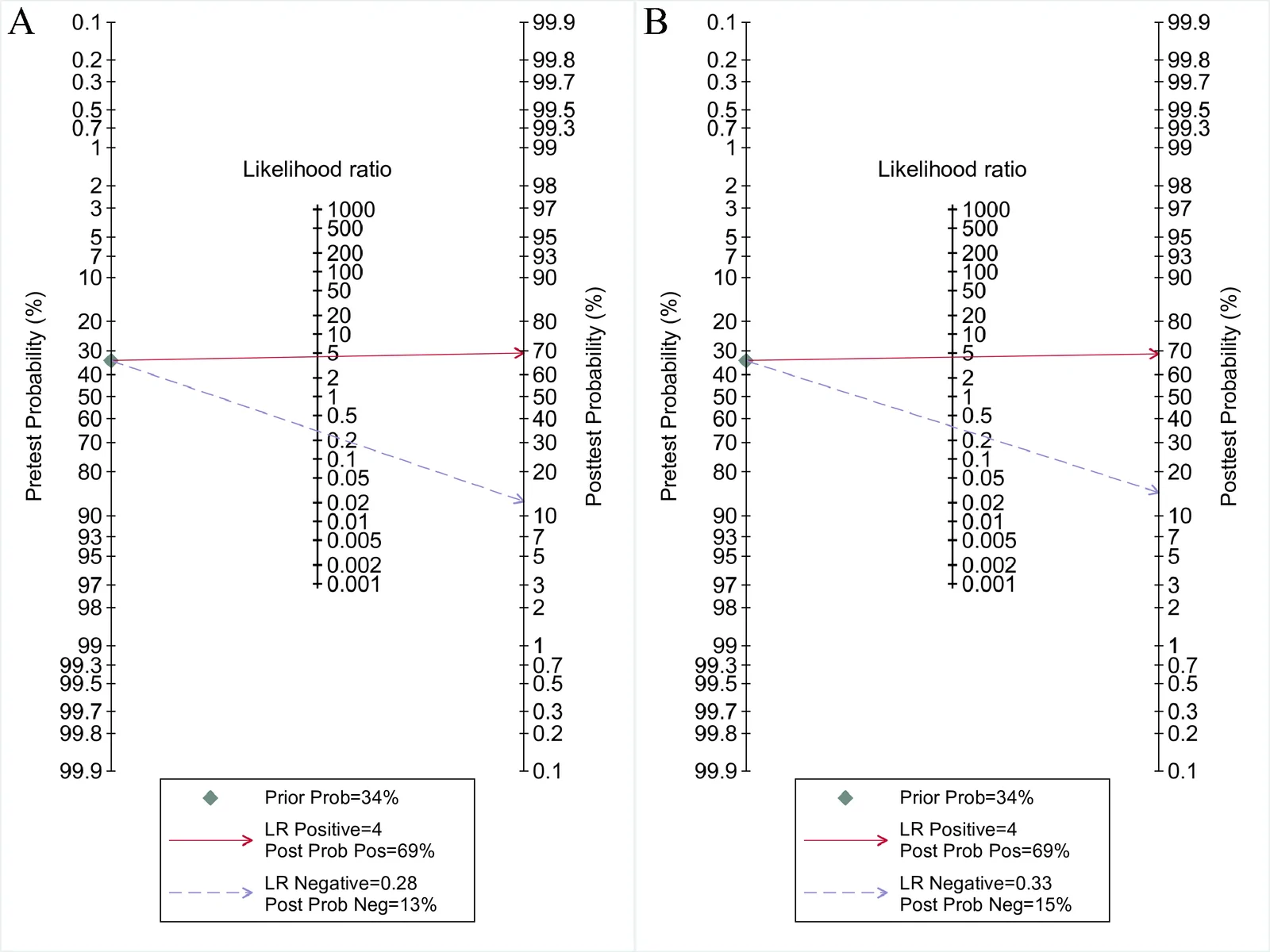
Fagan nomograms for estimating the probability of pathological complete response in the internal and external validation sets. (A) Fagan nomogram for the internal validation sets; (B) Fagan nomogram for the external validation sets. LR: likelihood ratio; Post Prob Neg: posttest probability after a negative result; Post Prob Pos: posttest probability after a positive result.

## Discussion

In this systematic review and meta-analysis, imaging-based AI models showed potentially useful diagnostic discrimination for pCR in patients with esophageal cancer undergoing neoadjuvant therapy, consistent with the biological information that radiomics features may capture. The AUC was numerically higher in internal (AUC=0.86) than in external (AUC=0.84) validation sets, although this difference was not statistically significant; sensitivity, specificity, and DOR were likewise comparable between cohorts. For the pooled sensitivity and specificity estimates, we also calculated PIs to quantify the distribution of effects across different populations and settings: the PIs were 0.57‐0.89 for sensitivity and 0.60‐0.93 for specificity in internal validation, and 0.34‐0.93 for sensitivity and 0.36‐0.98 for specificity in external validation (Figure S1 in Multimedia Appendix 1). The CIs quantify uncertainty in the average sensitivity and specificity; the PIs describe the range of true performance expected in a new study with conditions similar to those included. They do not describe individual patient probabilities or establish applicability in unrepresented regions. The wide PIs, high validation risk of bias in 12 of 33 studies [30,31,35,39,42-44,46,48,51,52,58], and very low certainty for all GRADE-assessed outcomes constrain the clinical interpretation. Although AUC estimates may vary with patient case mix, sample size, image acquisition parameters, and preprocessing workflows, the consistent direction of numerically higher internal-validation performance should be regarded as a possible signal of overfitting or limited generalizability rather than explained away as evidence of stable performance. Comparable sensitivity, specificity, and DOR estimates should therefore not be interpreted as resolving this concern. Statistical significance should likewise not be equated with clinical importance [60-62].

Subgroup analyses should be considered exploratory: only a limited number of datasets evaluated algorithms such as multilayer perceptron or Vision-Mamba, and the apparent differences may reflect small-study effects, dataset composition, or sampling variation rather than true algorithmic superiority (Table 4; Figure S3 in Multimedia Appendix 1). Conventional models such as LR and SVM can perform well in small, structured datasets, whereas newer DL architectures may capture more complex representations but generally require larger and more diverse datasets and may be more vulnerable to overfitting. In the current evidence base, the limited number of studies precludes firm conclusions about the comparative clinical advantages of these algorithms [63,64].

For different imaging modalities, no statistically significant differences were observed in sensitivity, specificity, or AUC between CT-based and MRI-based AI models for predicting pCR in esophageal cancer after neoadjuvant therapy. Although MRI-derived radiomics features can theoretically provide more detailed information on variations in tissue composition and tumor heterogeneity [65-67], the available evidence did not establish a reliable difference between the 2 modalities. This is primarily because the critical imaging signatures of tumor response are reflected in morphological changes, such as tumor volume reduction, and alterations in textural heterogeneity, which can be robustly captured by radiomics features derived from both modalities [60,68]. In addition, the standardization of radiomic features and the application of advanced learning methods may partly attenuate intrinsic differences between imaging modalities, resulting in comparable diagnostic performance. These findings do not establish equivalence or superior clinical utility of CT-based AI. Since CT remains the standard-of-care modality for staging and surveillance in esophageal cancer, characterized by lower cost, faster acquisition speed, and broader accessibility compared to MRI, routine CT workflows may offer a practical setting for further evaluation, but clinical benefit and cost-effectiveness remain unproven.

For patients receiving NACT, imaging-based AI models had the numerically highest pooled AUC (0.88, 95% CI 0.77‐0.98) among the treatment subgroups. This finding suggests that AI models might detect critical biological changes in tumors and their microenvironment before and after treatment. For both treatment modalities, NACT and NICT are able to remodel the tumor microenvironment and systemic inflammatory-immune status [69]. These dynamic changes can be reflected in clinical parameters such as the neutrophil-to-lymphocyte ratio (NLR), C-reactive protein (CRP), and hepatic function indices, all of which are closely associated with pCR [70-72].

In 2021, Kao and Hsu [18] reported a pooled AUC of 0.813 for radiomics-based prediction of pCR in esophageal cancer patients undergoing NCRT. Subsequently, Yang et al (2023) [11] observed an AUC of 0.84 in NCRT cohorts. Compared with the aforementioned studies, our study demonstrated comparable diagnostic performance, with AUCs of 0.86 in the internal validation set and 0.84 in the external validation set. Compared with the study by Kao and Hsu [18], our study evaluates AI model performance across multiple neoadjuvant treatment strategies, extending beyond NCRT, specifically including NICT and NACT. This expansion allows for a more comprehensive evaluation of AI models’ predictive potential across different therapeutic modalities. In contrast to the study by Yang et al (2023) [11], we separately extracted and synthesized results from internal and external validation cohorts, clarifying differences between the reported validation settings. In addition, we applied the latest PROBAST+AI tool for methodological quality assessment and the GRADE framework to evaluate the certainty of the evidence, making the limitations of the evidence explicit. To further explore the determinants of diagnostic performance, we conducted subgroup analyses based on imaging modalities (CT and MRI) and AI algorithms, alongside a sensitivity analysis focusing on ESCC. While these analyses provide deeper insights, the interstudy heterogeneity and the predominantly retrospective nature of the available evidence warrant cautious interpretation.

*I*² describes relative inconsistency in study results after allowing for sampling error, not the absolute magnitude of between-study heterogeneity. Because *I*² depends on within-study precision, its values should not be used to rank the amount of heterogeneity across outcomes or validation groups. Clinical interpretation instead relied on the PIs, which remained wide, particularly for external validation. A bivariate random-effects model accounted for between-study variation but could not eliminate study-level bias. Meta-regression and bivariate box plots were further conducted to explore potential sources of heterogeneity. The meta-regression indicated that the AI method and the type of AI model were likely the main contributors in the internal validation sets, whereas no statistically significant sources of heterogeneity were identified in the external validation sets. Radiomics extracts high-throughput features (intensity, shape, texture, and wavelets) reflecting tumor phenotype and microenvironment, which are complementary to clinical data [73-75]. The association between integrated radiomic and clinical models and specificity does not establish a causal reduction in misdiagnosis. These subgroup findings warrant cautious interpretation given the limited number of datasets. Moreover, bivariate box plots identified outliers, potentially contributing to statistical heterogeneity. Sensitivity analyses excluding low-quality studies or outliers and restricting to ESCC yielded similar average estimates under those tested conditions; they did not establish stability across new clinical settings.

Some limitations of the current meta-analysis should be considered when interpreting the results. First, most included studies adopted retrospective designs, which may introduce selection bias, information bias, and unmeasured confounding. Therefore, robust prospective studies are needed to validate these findings and assess applicability in clinical practice. Second, for each study, we extracted only the highest-performing AI algorithm to avoid within-study dependence among multiple candidate models. This selective extraction may produce overly optimistic performance estimates. The comparison using the first eligible imaging model reported in each article showed modest decreases in average performance but did not rule out selection bias. Third, all GRADE-assessed outcomes were of very low certainty, limiting the strength of the conclusions. A first-order limitation was geographic concentration: 32 of 33 studies [16,28-53,55-59] were conducted in China, with a strong predominance of ESCC. This concentration may reflect regional disease patterns, imaging practices, and publication patterns; the review could not determine their relative contributions. Differences in histological distributions, neoadjuvant treatment, CT and MRI protocols, and feature-extraction pipelines may limit transportability. External validation within this evidence base did not establish international generalizability. The English-only restriction may also have excluded relevant evidence. External validation in different geographic regions and ethnic populations is therefore required before widespread clinical implementation.

Our meta-analysis suggests that imaging-based AI models may provide useful predictive information across internal and external validation cohorts, but their performance and stability should be interpreted cautiously given the heterogeneity, geographic concentration, selective model extraction, and the very low certainty of the evidence. These models could serve as adjuncts to conventional radiological criteria; however, their clinical utility remains uncertain given the very low certainty of the evidence. The current evidence mainly concerns CT- or MRI-based models, sometimes combined with clinical variables; broader multimodal integration requires further evaluation. Therefore, in the future, integrating clinical imaging, pathology, and real-world data via AI models for multimodal dynamic analysis may improve esophageal cancer treatment response prediction [76]. Notably, AI remains an auxiliary tool, with clinical treatment decisions ultimately made by physicians according to each patient’s context. Furthermore, technical and regulatory barriers persist, and current AI algorithms still face multiple limitations in clinical practice, including data availability, model explainability, DL model generalization, and clinical integration. Advances in few-shot learning, self-supervised models, and centralized platforms may help build a more consolidated and transparent AI ecosystem, further enhancing clinicians’ decision-making and workflow efficiency [77,78].

In conclusion, by combining multiple neoadjuvant strategies and separating validation stages, this review extends earlier NCRT-focused syntheses and identifies priorities for further model evaluation. Imaging-based AI may support response assessment, but wide PIs, risk of bias, and very low evidence certainty preclude routine clinical adoption without prospective, geographically diverse validation.

## Supplementary material

10.2196/98520 Multimedia Appendix 1 Search strategies, risk-of-bias assessments, study characteristics, supplementary analyses, and figures.

10.2196/98520 Checklist 1 PRISMA-DTA checklist.

10.2196/98520 Checklist 2 PRISMA 2020 Abstracts checklist.

10.2196/98520 Checklist 3 PRISMA-S checklist.

## Abbreviations

AUC: area under the curve
CRP: C-reactive protein
CT: computed tomography
DL: deep learning
DOR: diagnostic odds ratio
ESCC: esophageal squamous cell carcinoma
ESMO: European Society for Medical Oncology
FN: false negative
FP: false positive
GRADE: Grading of Recommendations Assessment, Development, and Evaluation
HKSJ: Hartung-Knapp-Sidik-Jonkman
LR: logistic regression
LR+: positive likelihood ratio
LR-: negative likelihood ratio
ML: machine learning
MRI: magnetic resonance imaging
NACT: neoadjuvant chemotherapy
NCCN: National Comprehensive Cancer Network
NCRT: neoadjuvant chemoradiotherapy
NICRT: neoadjuvant immunotherapy plus chemoradiotherapy
NICT: neoadjuvant immunotherapy combined with chemotherapy
NLR: neutrophil-to-lymphocyte ratio
pCR: pathological complete response
PI: prediction interval
PITROS: participants, index test, target conditions, reference standard, outcomes, and settings
PRISMA: Preferred Reporting Items for Systematic Reviews and Meta-Analyses
PRISMA-DTA: Preferred Reporting Items for Systematic Reviews and Meta-Analyses for Diagnostic Test Accuracy
PRISMA-S: Preferred Reporting Items for Systematic Reviews and Meta-Analyses literature search extension
PROBAST+AI: Prediction Model Risk of Bias Assessment Tool plus Artificial Intelligence
PROSPERO: International Prospective Register of Systematic Reviews
RECIST: Response Evaluation Criteria in Solid Tumors
SROC: summary receiver operating characteristic curve
TN: true negative
TP: true positive
XGBoost: Extreme Gradient Boosting
