# Supplementary material for: Imaging-Based AI for Predicting Pathological Complete Response in Esophageal Cancer After Neoadjuvant Therapy: Systematic Review and Meta-Analysis
Source: J Med Internet Res. 2026 Nov 11;28:e98520. doi: 10.2196/98520 (PMC13754179; doi:10.2196/98520)
Supplement: Supplementary file 2 [file jmir-v28-e98520-s001.docx]

SUPPLEMENTARY MATERIALS

**Imaging-Based Artificial Intelligence for Predicting Pathological Complete Response in Esophageal Cancer After Neoadjuvant Therapy: A Systematic Review and Meta-Analysis**

Supplementary tables and figures accompanying the manuscript submitted to the Journal of Medical Internet Research.

10 Supplementary Tables · 6 Supplementary Figures

**Contents**

**Tables**

**Table S1.** Database search strategies	3

**Table S2.** PROBAST+AI: model development	5

**Table S3.** PROBAST+AI: model validation	8

**Table S4.** Original model characteristics and fourfold counts	11

**Table S5.** CT acquisition details	14

**Table S6.** MRI acquisition details	17

**Table S7.** Meta-regression: internal validation	18

**Table S8.** Meta-regression: external validation	19

**Table S9.** First reported eligible models	20

**Table S10.** Matched model-selection sensitivity analysis	22

**Figures**

**Figure S1.** Sensitivity and specificity forest plots	23

**Figure S2.** SROC curves and joint regions	25

**Figure S3.** Algorithm-specific estimates	26

**Figure S4.** Sensitivity analysis: excluding outliers	27

**Figure S5.** Sensitivity analysis: excluding low-quality studies	28

**Figure S6.** Sensitivity analysis: ESCC only	29

### **Table S1.** Search strategy in PubMed, Embase, Cochrane and Web of Science.

| **Database** | **Search strategy** | **Filters and Limits** | **Number of Studies** |
| --- | --- | --- | --- |
| PubMed | ("Esophageal Neoplasms"[Mesh] OR "Esophageal Squamous Cell Carcinoma"[Mesh] OR esophag*[tiab] OR oesophag*[tiab] OR ESCC[tiab] OR EAC[tiab]) AND ("Radiomics"[Mesh] OR "Artificial Intelligence"[Mesh] OR "Machine Learning"[Mesh] OR "Deep Learning"[Mesh] OR "Neural Networks, Computer"[Mesh] OR "Image Processing, Computer-Assisted"[Mesh] OR radiomic*[tiab] OR "delta radiomics"[tiab] OR "delta-radiomics"[tiab] OR "delta radiomic"[tiab] OR "texture analysis"[tiab] OR "texture analyses"[tiab] OR "textural analysis"[tiab] OR "quantitative imaging"[tiab] OR "imaging biomarker"[tiab] OR "imaging biomarkers"[tiab] OR "artificial intelligence"[tiab] OR "machine learning"[tiab] OR "deep learning"[tiab] OR "neural network"[tiab] OR "neural networks"[tiab] OR "deep neural network"[tiab] OR "deep neural networks"[tiab] OR "convolutional neural network"[tiab] OR "convolutional neural networks"[tiab] OR CNN[tiab] OR "support vector machine"[tiab] OR "support vector machines"[tiab] OR SVM[tiab] OR "random forest"[tiab] OR "random forests"[tiab] OR "gradient boosting"[tiab] OR XGBoost[tiab] OR LASSO[tiab] OR "least absolute shrinkage and selection operator"[tiab] OR "elastic net"[tiab] OR "prediction model"[tiab] OR "prediction models"[tiab] OR nomogram*[tiab] OR "radiomics signature"[tiab] OR "radiomic signature"[tiab] OR "radiomics nomogram"[tiab] OR "radiomic nomogram"[tiab]) AND ("Neoadjuvant Therapy"[Mesh] OR neoadjuvant*[tiab] OR "preoperative chemotherapy"[tiab] OR "pre-operative chemotherapy"[tiab] OR "preoperative chemoradiotherapy"[tiab] OR "pre-operative chemoradiotherapy"[tiab] OR "preoperative chemoradiation"[tiab] OR "pre-operative chemoradiation"[tiab] OR "preoperative radiotherapy"[tiab] OR "pre-operative radiotherapy"[tiab] OR "preoperative immunotherapy"[tiab] OR "pre-operative immunotherapy"[tiab] OR "induction chemotherapy"[tiab] OR "induction therapy"[tiab] OR trimodality[tiab] OR "trimodality therapy"[tiab] OR "total neoadjuvant therapy"[tiab] OR NACT[tiab] OR NAC[tiab] OR nCRT[tiab] OR nICT[tiab] OR nICRT[tiab] OR TNT[tiab]) | No restrictions applied regarding date range, language, or publication type. | 411 |
| Embase | ('esophageal cancer'/exp OR 'esophageal squamous cell carcinoma'/exp OR esophag*:ti,ab,kw OR oesophag*:ti,ab,kw OR escc:ti,ab,kw OR eac:ti,ab,kw) AND ('radiomics'/exp OR 'artificial intelligence'/exp OR 'machine learning'/exp OR 'deep learning'/exp OR 'neural network'/exp OR 'image processing'/exp OR radiomic*:ti,ab,kw OR 'delta radiomics':ti,ab,kw OR 'delta-radiomics':ti,ab,kw OR 'delta radiomic':ti,ab,kw OR 'texture analysis':ti,ab,kw OR 'texture analyses':ti,ab,kw OR 'textural analysis':ti,ab,kw OR 'quantitative imaging':ti,ab,kw OR 'imaging biomarker':ti,ab,kw OR 'imaging biomarkers':ti,ab,kw OR 'artificial intelligence':ti,ab,kw OR 'machine learning':ti,ab,kw OR 'deep learning':ti,ab,kw OR 'neural network':ti,ab,kw OR 'neural networks':ti,ab,kw OR 'deep neural network':ti,ab,kw OR 'deep neural networks':ti,ab,kw OR 'convolutional neural network':ti,ab,kw OR 'convolutional neural networks':ti,ab,kw OR cnn:ti,ab,kw OR 'support vector machine':ti,ab,kw OR 'support vector machines':ti,ab,kw OR svm:ti,ab,kw OR 'random forest':ti,ab,kw OR 'random forests':ti,ab,kw OR 'gradient boosting':ti,ab,kw OR xgboost:ti,ab,kw OR lasso:ti,ab,kw OR 'least absolute shrinkage and selection operator':ti,ab,kw OR 'elastic net':ti,ab,kw OR 'prediction model':ti,ab,kw OR 'prediction models':ti,ab,kw OR nomogram*:ti,ab,kw OR 'radiomics signature':ti,ab,kw OR 'radiomic signature':ti,ab,kw OR 'radiomics nomogram':ti,ab,kw OR 'radiomic nomogram':ti,ab,kw) AND ('neoadjuvant therapy'/exp OR neoadjuvant*:ti,ab,kw OR 'preoperative chemotherapy':ti,ab,kw OR 'pre-operative chemotherapy':ti,ab,kw OR 'preoperative chemoradiotherapy':ti,ab,kw OR 'pre-operative chemoradiotherapy':ti,ab,kw OR 'preoperative chemoradiation':ti,ab,kw OR 'pre-operative chemoradiation':ti,ab,kw OR 'preoperative radiotherapy':ti,ab,kw OR 'pre-operative radiotherapy':ti,ab,kw OR 'preoperative immunotherapy':ti,ab,kw OR 'pre-operative immunotherapy':ti,ab,kw OR 'induction chemotherapy':ti,ab,kw OR 'induction therapy':ti,ab,kw OR trimodality:ti,ab,kw OR 'trimodality therapy':ti,ab,kw OR 'total neoadjuvant therapy':ti,ab,kw OR nact:ti,ab,kw OR nac:ti,ab,kw OR ncrt:ti,ab,kw OR nict:ti,ab,kw OR nicrt:ti,ab,kw OR tnt:ti,ab,kw) | No restrictions applied regarding date range, language, or publication type. | 734 |
| Web of Science | TS=(esophag* OR oesophag* OR ESCC OR EAC) AND TS=(radiomic* OR "delta radiomics" OR "delta-radiomics" OR "delta radiomic" OR "texture analysis" OR "texture analyses" OR "textural analysis" OR "quantitative imaging" OR "imaging biomarker" OR "imaging biomarkers" OR "artificial intelligence" OR "machine learning" OR "deep learning" OR "neural network" OR "neural networks" OR "deep neural network" OR "deep neural networks" OR "convolutional neural network" OR "convolutional neural networks" OR CNN OR "support vector machine" OR "support vector machines" OR SVM OR "random forest" OR "random forests" OR "gradient boosting" OR XGBoost OR LASSO OR "least absolute shrinkage and selection operator" OR "elastic net" OR "prediction model" OR "prediction models" OR nomogram* OR "radiomics signature" OR "radiomic signature" OR "radiomics nomogram" OR "radiomic nomogram") AND TS=(neoadjuvant* OR "preoperative chemotherapy" OR "pre-operative chemotherapy" OR "preoperative chemoradiotherapy" OR "pre-operative chemoradiotherapy" OR "preoperative chemoradiation" OR "pre-operative chemoradiation" OR "preoperative radiotherapy" OR "pre-operative radiotherapy" OR "preoperative immunotherapy" OR "pre-operative immunotherapy" OR "induction chemotherapy" OR "induction therapy" OR trimodality OR "trimodality therapy" OR "total neoadjuvant therapy" OR NACT OR NAC OR nCRT OR nICT OR nICRT OR TNT) | No restrictions applied regarding date range, language, or publication type. | 521 |
| Cochrane Library | (esophagus OR esophageal OR oesophagus OR oesophageal OR "esophageal cancer" OR "oesophageal cancer" OR "esophageal carcinoma" OR "oesophageal carcinoma" OR "esophageal neoplasm" OR "oesophageal neoplasm" OR "esophageal tumor" OR "esophageal tumors" OR "oesophageal tumour" OR "oesophageal tumours" OR "esophageal adenocarcinoma" OR "oesophageal adenocarcinoma" OR "esophageal squamous cell carcinoma" OR "oesophageal squamous cell carcinoma" OR ESCC OR EAC):ti,ab,kw AND (radiomic OR radiomics OR "radiomic-based" OR "radiomics-based" OR "delta radiomic" OR "delta radiomics" OR "delta-radiomic" OR "delta-radiomics" OR "texture analysis" OR "texture analyses" OR "textural analysis" OR "quantitative imaging" OR "imaging biomarker" OR "imaging biomarkers" OR "artificial intelligence" OR "machine learning" OR "deep learning" OR "neural network" OR "neural networks" OR "deep neural network" OR "deep neural networks" OR "convolutional neural network" OR "convolutional neural networks" OR CNN OR "support vector machine" OR "support vector machines" OR SVM OR "random forest" OR "random forests" OR "gradient boosting" OR XGBoost OR LASSO OR "least absolute shrinkage and selection operator" OR "elastic net" OR "prediction model" OR "prediction models" OR "predictive model" OR "predictive models" OR nomogram OR nomograms OR "radiomic signature" OR "radiomics signature" OR "radiomic signatures" OR "radiomics signatures" OR "radiomic nomogram" OR "radiomics nomogram" OR "radiomic nomograms" OR "radiomics nomograms"):ti,ab,kw AND (neoadjuvant OR "neoadjuvant therapy" OR "neoadjuvant treatment" OR "neoadjuvant chemotherapy" OR "neoadjuvant chemoradiotherapy" OR "neoadjuvant chemoradiation" OR "neoadjuvant radiochemotherapy" OR "neoadjuvant radiotherapy" OR "neoadjuvant immunotherapy" OR "neoadjuvant immunochemotherapy" OR "neoadjuvant chemoimmunotherapy" OR "neoadjuvant systemic therapy" OR "neoadjuvant systemic treatment" OR "preoperative chemotherapy" OR "pre-operative chemotherapy" OR "preoperative chemoradiotherapy" OR "pre-operative chemoradiotherapy" OR "preoperative chemoradiation" OR "pre-operative chemoradiation" OR "preoperative radiochemotherapy" OR "pre-operative radiochemotherapy" OR "preoperative radiotherapy" OR "pre-operative radiotherapy" OR "preoperative immunotherapy" OR "pre-operative immunotherapy" OR "induction chemotherapy" OR "induction therapy" OR trimodality OR "trimodality therapy" OR "total neoadjuvant therapy" OR NACT OR NAC OR nCRT OR nICT OR nICRT OR TNT):ti,ab,kw | No restrictions applied regarding date range, language, or publication type. | 14 |

### **Table S2.** Risk of bias assessment (PROBAST+AI) model development based on seven domains for training Set.

| **Author, year** | | **Quality** | | | | **Applicability concerns** | | | **Overall judgement** | |
| --- | --- | --- | --- | --- | --- | --- | --- | --- | --- | --- |
|  |  | **Participants and data sources ^a^** | **Predictors ^b^** | **Outcome ^c^** | **Analysis ^d^** | **Participants and data sources ^e^** | **Predictors ^f^** | **Outcome ^g^** | **Quality ^h^** | **Applicability concerns ^i^** |
| J Wang et al. | 2023 | L | L | L | L | L | L | L | L | L |
| J Ou et al. | 2024 | L | L | L | L | L | L | L | L | L |
| X Wang et al. | 2024 | L | L | L | L | L | L | L | L | L |
| Z Zhang et al. | 2025 | L | L | L | L | L | L | L | L | L |
| Y Qi et al. | 2025 | L | L | L | L | L | L | L | L | L |
| Y Zheng et al. | 2024 | L | L | L | L | L | L | L | L | L |
| M Zhang et al. | 2023 | L | L | L | L | L | L | L | L | L |
| W-X Qi et al. | 2024 | U | L | U | L | L | L | U | U | U |
| Z Lu et al. | 2024 | L | L | L | L | L | L | L | L | L |
| Y Liu et al. | 2024 | L | L | L | L | L | L | L | L | L |
| Y Liu et al. | 2024 | L | L | L | L | L | L | L | L | L |
| W Kong et al. | 2024 | L | L | L | L | L | L | L | L | L |
| L Fan et al. | 2024 | L | L | L | L | L | L | L | L | L |
| Z Li et al. | 2024 | L | L | L | L | L | L | L | L | L |
| K Li et al. | 2023 | L | L | L | L | L | L | L | L | L |
| K Li et al. | 2023 | L | L | U | L | L | L | U | U | U |
| H Yue et al. | 2022 | L | L | U | L | L | L | U | U | U |
| Y Li et al. | 2021 | L | L | L | L | L | L | L | L | L |
| Y Hu et al. | 2020 | L | L | L | L | L | L | L | L | L |
| Z Yang et al. | 2019 | L | L | L | H | L | L | L | H | L |
| J Zhu et al. | 2025 | L | L | L | L | L | L | L | L | L |
| Y Sun et al. | 2024 | U | L | L | L | L | L | L | U | L |
| Q Gu et al. | 2025 | L | L | L | L | L | L | L | L | L |
| A Hirata et al. | 2025 | U | L | L | L | H | L | L | U | H |
| [X Ling](https://pubmed.ncbi.nlm.nih.gov/?term=) et al. | 2025 | L | L | L | L | L | L | L | L | L |
| L Liu et al. | 2025 | L | L | L | L | L | L | L | L | L |
| Y Liu et al. | 2025 | L | L | L | L | L | L | L | L | L |
| B Qi et al. | 2025 | L | L | L | L | L | L | L | L | L |
| [L Shi](https://pubmed.ncbi.nlm.nih.gov/?term=) et al. | 2026 | U | L | L | L | L | L | L | U | L |
| Z Xu et al. | 2026 | L | L | U | L | L | L | U | U | U |
| B Zhao et al. | 2026 | L | L | L | L | L | L | L | L | L |
| Z Zhao et al. | 2025 | L | L | L | L | L | L | L | L | L |
| B Xu et al. | 2025 | L | L | U | L | L | L | U | U | U |

**A**bbreviation: PROBAST+AI, Prediction model Risk of Bias Assessment Tool plus Artificial Intelligence, L low; H high; U unclear.

**Footnote: Signaling questions are rated as "yes" (Y), "probably yes" (PY), "probably no" (PN), "no" (N), "no information" (NI), and in some cases "not applicable" (NA). All signaling questions are phrased in such a way that "yes" or "probably yes" indicates a low risk of bias. Any signaling questions rated as "no" or "probably no" indicate a potential high risk of bias in that domain. If there are no "no" or "probably no" ratings, but "no information" (NI) is present, the risk of bias in that domain is classified as unclear.**

a. Participants and data sources

1.1 Were appropriate data sources used?

1.2 Was an appropriate study design used?

1.3 Did the in- and exclusions of study participants result in a representative dataset?

b. Predictors

2.1 Were predictors defined and assessed in a similar way for all participants?

2.2 Was any pre-processing of predictors similar for all participants?

2.3 Were predictor assessments made without knowledge of outcome data?

2.4 Were the predictors included in the model available at the time the model was intended to be used?

c. Outcome

3.1 Were outcomes defined and assessed appropriately?

3.2 Were outcomes defined and assessed in a similar way for all participants?

3.3 Were outcome assessments made without use or knowledge of predictor data?

3.4 Was the time interval between predictor assessment and outcome assessment appropriate?

d. Analysis

4.1 Was there evidence that the sample size was reasonable?

4.2 Were continuous and categorical predictors handled appropriately?

4.3 Were participants with missing or censored data handled appropriately in the analysis?

4.4 If methods to address class imbalance were used, was the model or the model predictions recalibrated?

4.5 Were methods used to address potential model overfitting?

e. Participants and data sources

Concern that the (data of the) included participants do not match the review question or the assessor’s intended use of the prediction model.

f. Predictors

Concern that the definition, pre-processing, assessment, or timing of assessment of the predictors in the model do not match the review question or the assessor’s intended use.

g. Outcome

Concern that the outcome, its definition, assessment, or timing of assessment do not match the review question or the assessor’s intended use.

h. Quality

Low risk: If all four domains were rated low concern regarding quality.

High risk: If at least one domain was rated high concern regarding quality.

Unclear: If at least one domain was rated unclear concern regarding quality and no domains were rated high concern.

i. Applicability concerns

Low risk: If all three domains were rated low concern for applicability.

High risk: If at least one domain was rated high concern for applicability.

Unclear: If at least one domain was rated unclear concern for applicability and no domains were rated high concern.

### **Table S3.** Risk of bias assessment (PROBAST+AI) model evaluation based on seven domains for validation set.

| **Author, year** | | **Risk of bias** | | | | **Applicability concerns** | | | **Overall judgement** | |
| --- | --- | --- | --- | --- | --- | --- | --- | --- | --- | --- |
|  |  | **Participants and data sources ^a^** | **Predictors ^b^** | **Outcome ^c^** | **Analysis ^d^** | **Participants and data sources ^e^** | **Predictors ^f^** | **Outcome ^g^** | **Risk of bias ^h^** | **Applicability concerns ^i^** |
| J Wang et al. | 2023 | L | L | L | L | L | L | L | L | L |
| J Ou et al. | 2024 | L | L | L | L | L | L | L | L | L |
| X Wang et al. | 2024 | L | L | L | L | L | L | L | L | L |
| Z Zhang et al. | 2025 | L | L | L | L | L | L | L | L | L |
| Y Qi et al. | 2025 | L | L | L | L | L | L | L | L | L |
| Y Zheng et al. | 2024 | L | L | L | H | L | L | L | H | L |
| M Zhang et al. | 2023 | L | L | L | H | L | L | L | H | L |
| W-X Qi et al. | 2024 | U | L | U | H | L | L | U | H | U |
| Z Lu et al. | 2024 | L | L | L | H | L | L | L | H | L |
| Y Liu et al. | 2024 | L | L | L | L | L | L | L | L | L |
| Y Liu et al. | 2024 | L | L | L | H | L | L | L | H | L |
| W Kong et al. | 2024 | L | L | L | H | L | L | L | H | L |
| L Fan et al. | 2024 | L | L | L | H | L | L | L | H | L |
| Z Li et al. | 2024 | L | L | L | L | L | L | L | L | L |
| K Li et al. | 2023 | L | L | L | L | L | L | L | L | L |
| K Li et al. | 2023 | L | L | U | H | L | L | L | H | L |
| H Yue et al. | 2022 | L | L | U | L | L | L | U | U | U |
| Y Li et al. | 2021 | L | L | L | L | L | L | L | L | L |
| Y Hu et al. | 2020 | L | L | L | L | L | L | L | L | L |
| Z Yang et al. | 2019 | L | L | L | H | L | L | L | H | L |
| J Zhu et al. | 2025 | L | L | L | L | L | L | L | L | L |
| Y Sun et al. | 2024 | U | L | L | H | L | L | L | H | L |
| Q Gu et al. | 2025 | L | L | L | L | L | L | L | L | L |
| A Hirata et al. | 2025 | U | L | L | L | H | L | L | U | H |
| [X Ling](https://pubmed.ncbi.nlm.nih.gov/?term=) et al. | 2025 | L | L | L | H | L | L | L | H | L |
| L Liu et al. | 2025 | L | L | L | L | L | L | L | L | L |
| Y Liu et al. | 2025 | L | L | L | L | L | L | L | L | L |
| B Qi et al. | 2025 | L | L | L | L | L | L | L | L | L |
| [L Shi](https://pubmed.ncbi.nlm.nih.gov/?term=) et al. | 2026 | U | L | L | L | L | L | L | U | L |
| Z Xu et al. | 2026 | L | L | U | L | L | L | U | U | U |
| B Zhao et al. | 2026 | L | L | L | H | L | L | L | H | L |
| Z Zhao et al. | 2025 | L | L | L | L | L | L | L | L | L |
| B Xu et al. | 2025 | L | L | U | L | L | L | U | U | U |

**A**bbreviation: PROBAST+AI, Prediction model Risk of Bias Assessment Tool plus Artificial Intelligence, L low; H high; U unclear.

**Footnote: Signaling questions are rated as "yes" (Y), "probably yes" (PY), "probably no" (PN), "no" (N), "no information" (NI), and in some cases "not applicable" (NA). All signaling questions are phrased in such a way that "yes" or "probably yes" indicates a low risk of bias. Any signaling questions rated as "no" or "probably no" indicate a potential high risk of bias in that domain. If there are no "no" or "probably no" ratings, but "no information" (NI) is present, the risk of bias in that domain is classified as unclear.**

a. Participants and data sources

1.1 Were appropriate data sources used?

1.2 Was an appropriate study design used?

1.3 Did the in- and exclusions of study participants result in a representative dataset?

b. Predictors

2.1 Were predictors defined and assessed in a similar way for all participants?

2.2 Was any pre-processing of predictors similar for all participants?

2.3 Were predictor assessments made without knowledge of outcome data?

2.4 Were the predictors included in the model available at the time the model was intended to be used?

c. Outcome

3.1 Were outcomes defined and assessed appropriately?

3.2 Were outcomes defined and assessed in a similar way for all participants?

3.3 Were outcome assessments made without use or knowledge of predictor data?

3.4 Was the time interval between predictor assessment and outcome assessment appropriate?

d. Analysis

4.1 Was model evaluation based on only apparent performance avoided?

4.2 Was there evidence that the sample size was reasonable?

4.3 Were participants with missing or censored data handled appropriately in the analysis?

4.4 If methods to address class imbalance were used, was the evaluation done in a dataset without imbalance correction?

4.5 If data splitting was done to create training and test datasets, was there evidence that data leakage was avoided?

4.6 If resampling methods were used to evaluate model performance, were all model development steps replicated in the resampling process?

4.7 Was the predictive performance of the model evaluated appropriately, e.g., calibration, discrimination, and net benefit?

e. Participants and data sources

Concern that the (data of the) included participants do not match the review question or the assessor’s intended use of the prediction model.

f. Predictors

Concern that the definition, pre-processing, assessment, or timing of assessment of the predictors in the model do not match the review question or the assessor’s intended use.

g. Outcome

Concern that the outcome, its definition, assessment, or timing of assessment do not match the review question or the assessor’s intended use.

h. Risk of bias

Low risk: If all four domains were rated low risk of bias.

High risk: If at least one domain was rated high risk of bias.

Unclear: If at least one domain was rated unclear risk of bias and no domains were rated high risk of bias.

i. Applicability concerns

Low risk: If all three domains were rated low concern for applicability.

High risk: If at least one domain was rated high concern for applicability.

Unclear: If at least one domain was rated unclear concern for applicability and no domains were rated high concern.

### **Table S4.** Technical aspects of included studies.

| **Author** | **Year** | **Type of imaging** | **Data source** | **AI model** | **Data splitting method** | **AI method** | **AI algorithms** | **Internal validation** | | | | | **External validation** | | | | |
| --- | --- | --- | --- | --- | --- | --- | --- | --- | --- | --- | --- | --- | --- | --- | --- | --- | --- |
|  |  |  |  |  |  |  |  | **TP** | **FP** | **FN** | **TN** | **AUC** | **TP** | **FP** | **FN** | **TN** | **AUC** |
| J Wang et al. | 2023 | CT | Local hospital | Radiomic & clinical model | LOOCV | ML | LR | 32 | 19 | 16 | 45 | 0.81 | NA | NA | NA | NA | NA |
| J Ou et al. | 2024 | CT | Local hospital | Radiomic model | Hold-out | ML | SVM | 25 | 8 | 4 | 46 | 0.87 | 16 | 9 | 5 | 25 | 0.87 |
| X Wang et al. | 2024 | CT | Local hospital | Radiomic & clinical model | 5-fold cross-validation | ML | XGBoost | 22 | 2 | 6 | 30 | 0.84 | NA | NA | NA | NA | NA |
| Z Zhang et al. | 2025 | CT | Local hospital | Radiomic model | Hold-out | DL | Vision-Mamba | 14 | 11 | 4 | 89 | 0.83 | 8 | 0 | 3 | 23 | 0.86 |
| Y Qi et al. | 2025 | CT | Local hospital | Radiomic model | Hold-out | ML | SVM | 6 | 6 | 9 | 33 | 0.70 | NA | NA | NA | NA | NA |
| Y Zheng et al. | 2024 | CT | Local hospital | Radiomic model | Independent dataset validation | ML | LR | NA | NA | NA | NA | NA | 10 | 10 | 3 | 24 | 0.79 |
| M Zhang et al. | 2023 | CT | Local hospital | Radiomic model | Hold-out | ML | XGBoost | 14 | 9 | 2 | 21 | 0.84 | 9 | 6 | 3 | 15 | 0.79 |
| W-X Qi et al. | 2024 | CT | Local hospital | Radiomic model | Hold-out | ML | SVM | 11 | 6 | 2 | 7 | 0.78 | NA | NA | NA | NA | NA |
| Z Lu et al. | 2024 | CT | Local hospital | Radiomic & clinical model | Hold-out | ML | LR | 7 | 2 | 4 | 18 | 0.85 | NA | NA | NA | NA | NA |
| Y Liu et al. | 2024 | MRI | Local hospital | Radiomic model | Independent dataset validation | ML | RF | NA | NA | NA | NA | NA | 18 | 7 | 6 | 42 | 0.89 |
| Y Liu et al. | 2024 | MRI | Local hospital | Radiomic & clinical model | Hold-out | ML | LR | 10 | 2 | 2 | 11 | 0.90 | NA | NA | NA | NA | NA |
| W Kong et al. | 2024 | CT | Local hospital | Radiomic & clinical model | Independent dataset validation | ML | LR | NA | NA | NA | NA | NA | 3 | 2 | 3 | 19 | 0.85 |
| L Fan et al. | 2024 | CT | Local hospital | Radiomic & clinical model | 5-fold cross-validation | ML | LR | 16 | 5 | 1 | 24 | 0.96 | NA | NA | NA | NA | NA |
| Z Li et al. | 2024 | CT | Local hospital | Radiomic model | Hold-out | ML | RF | 13 | 15 | 1 | 33 | 0.87 | 8 | 4 | 4 | 14 | 0.75 |
| K Li et al. | 2023 | CT | Local hospital | Radiomic & clinical model | Independent dataset validation | ML | LR | NA | NA | NA | NA | NA | 3 | 3 | 1 | 20 | 0.90 |
| K Li et al. | 2023 | CT | Local hospital | Radiomic model | Hold-out | ML | RF | 8 | 2 | 4 | 15 | 0.85 | NA | NA | NA | NA | NA |
| H Yue et al. | 2022 | CT | Local hospital | Radiomic model | 5-fold cross-validation | DL | MLP | 43 | 24 | 6 | 51 | 0.87 | NA | NA | NA | NA | NA |
| Y Li et al. | 2021 | CT | Local hospital | Radiomic & clinical model | Bootstrapping | ML | LR | 37 | 7 | 14 | 63 | 0.84 | NA | NA | NA | NA | NA |
| Y Hu et al. | 2020 | CT | Local hospital | Radiomic model | Independent dataset validation | ML | SVM | NA | NA | NA | NA | NA | 28 | 8 | 3 | 31 | 0.85 |
| Z Yang et al. | 2019 | CT | Local hospital | Radiomic model | Hold-out | ML | LR | 3 | 1 | 1 | 6 | 0.79 | NA | NA | NA | NA | NA |
| J Zhu et al. | 2025 | CT | Local hospital | Radiomic model | Hold-out | ML | XGBoost | 16 | 9 | 4 | 40 | 0.83 | 10 | 6 | 2 | 23 | 0.79 |
| Y Sun et al. | 2024 | CT | Local hospital | Radiomic model | Hold-out | DL | T2T-ViT | 12 | 4 | 2 | 16 | 0.89 | NA | NA | NA | NA | NA |
| Q Gu et al. | 2025 | CT | Local hospital | Radiomic model | Time-Series Split | DL | FCN | 31 | 17 | 9 | 196 | 0.95 | 18 | 5 | 2 | 15 | 0.93 |
| A Hirata et al. | 2025 | MRI | Local hospital | Radiomic model | Nested Cross-Validation | ML | KNN | 12 | 8 | 3 | 47 | 0.85 | NA | NA | NA | NA | NA |
| [X Ling](https://pubmed.ncbi.nlm.nih.gov/?term=) et al. | 2025 | CT | Local hospital | Radiomic & clinical model | Time-Series Split | ML | XGBoost | NA | NA | NA | NA | NA | 7 | 1 | 1 | 38 | 0.96 |
| L Liu et al. | 2025 | CT | Local hospital | Radiomic model | LOOCV | ML | XGBoost | 14 | 3 | 4 | 45 | 0.90 | NA | NA | NA | NA | NA |
| Y Liu et al. | 2025 | CT | Local hospital | Radiomic model | Independent dataset validation | DL | 3D CNN | 8 | 2 | 10 | 29 | 0.80 | 13 | 7 | 6 | 32 | 0.83 |
| B Qi et al. | 2025 | CT | Local hospital | Radiomic model | Independent dataset validation | ML | XGBoost | 13 | 28 | 8 | 66 | 0.74 | 7 | 10 | 6 | 16 | 0.68 |
| [L Shi](https://pubmed.ncbi.nlm.nih.gov/?term=) et al. | 2026 | CT | Local hospital | Radiomic & clinical model | Hold-out | ML | LR | 37 | 13 | 7 | 37 | 0.84 | NA | NA | NA | NA | NA |
| Z Xu et al. | 2026 | CT | Local hospital | Radiomic model | Independent dataset validation | ML | ExtraTrees | 17 | 4 | 5 | 21 | 0.90 | 2 | 1 | 17 | 61 | 0.84 |
| B Zhao et al. | 2026 | CT | Local hospital | Radiomic & clinical model | Hold-out | ML | LR | 8 | 4 | 7 | 22 | 0.86 | NA | NA | NA | NA | NA |
| Z Zhao et al. | 2025 | CT | Local hospital | Radiomic model | Independent dataset validation | DL | MLP | 9 | 10 | 3 | 18 | 0.69 | 18 | 36 | 4 | 17 | 0.60 |
| B Xu et al. | 2025 | CT | Local hospital | Radiomic model | Hold-out | ML | LR | 17 | 13 | 3 | 32 | 0.80 | NA | NA | NA | NA | NA |

ML Machine learning; DL Deep learning; CT Computed Tomography; MRI Magnetic Resonance Imaging; TP true positive; TN true negative; FP false positive; FN false negative; NA not available; LR Logistic Regression;SVM Support Vector Machines;RF Random Forest; MLP multi-layer perceptron; XGBoost Extreme Gradient Boosting; CNN convolutional neural network; T2T-ViT token-to-token Vision Transformer; FCN Fully Convolutional Network; KNN K-Nearest Neighbors.

### **Table S5.** Technical aspects of included studies for CT

| **Author** | **Year** | **Scanner Modality (Manufacturer)** | **Evaluation Time** | **Regions of interest (ROI)** | **Enhancement phase** | **Contrast-enhanced** |
| --- | --- | --- | --- | --- | --- | --- |
| J Wang et al. | 2023 | Philips Brilliance CT Big Bore Oncology Configuration (Philips) | Pre-treatment | Manually | Single-phase-contrast-  enhanced | Yes |
| J Ou et al. | 2024 | LightSpeed VCT (GE) and SOMATOM Definition Flash (Siemens) | Pre-treatment & post-treatment | Manually | Arterial phase, portal venous phase, and delay phase | Yes |
| X Wang et al. | 2024 | SOMATOM Deﬁnition AS+ (Siemens) | Pre-treatment & post-treatment | Manually | Arterial phase and venous phase | Yes |
| Z Zhang et al. | 2025 | Optima CT680 (GE), Definition Flash (Siemens), Ingenuity CT (Philips), Optima CT680 Expert (GE), Revolution EVO (GE), SOMATOM Definition AS+ (Siemens), SOMATOM Drive (Siemens), IQon - Spectral (Philips), BrightSpeed (GE), LightSpeed VCT (GE), and Optima CT680 Series(GE) | Pre-treatment | Manually | NA | Yes |
| Y Qi et al. | 2025 | NA (Siemens) | Pre-treatment | Manually | Arterial phase | Yes |
| Y Zheng et al. | 2024 | Light Speed VCT (GE), SOMATOM Definition (Siemens), and SOMATOM Definition AS+ (Siemens) | Pre-treatment & post-treatment | Semi-automatically | NA | Yes |
| M Zhang et al. | 2023 | NA (Siemens) | Pre-treatment & post-treatment | Manually | NA | Yes |
| W-X Qi et al. | 2024 | NA | pre-treatment | Manually | NA | Yes |
| Z Lu et al. | 2024 | Lightspeed 64-slice spiral CT scanner (GE) | Pre-treatment & post-treatment | Manually | Late arterial phase | Yes |
| W Kong et al. | 2024 | Revolution CT (GE) | Pre-treatment | Manually | Artery phase and venous phase | Yes |
| L Fan et al. | 2024 | iCT 128 (Philips) | Pre-treatment & post-treatment | Semi-automatically | NA | NA |
| Z Li et al. | 2024 | GE, Siemens, Philips | Pre-treatment | Manually | NA | Yes |
| K Li et al. | 2023 | Aquilion TSX-101A (Toshiba Medical Systems), Discovery 750 HD (GE), Brightspeed (GE), and Ingenuity (Philips), Definition Flash (Siemens) | Pre-treatment & post-treatment | Manually | Artery phase | Yes |
| K Li et al. | 2023 | SOMATOM Force (Siemens), iCT 256 (Philips), MEDICAL SYSTEMS Revolution CT (GE), NMS NeuViz Glory (Neusoft Medical Systems), MinFound ScintCareCT 128 (MinFound Medical Systems), TOSHIBA (Canon Medical Systems) | Pre-treatment & post-treatment | Manually | Artery phase | Yes |
| H Yue et al. | 2022 | NA | Pre-treatment & post-treatment | Manually | NA | NA |
| Y Li et al. | 2021 | NA | Pre-treatment & post-treatment | Manually | NA | Yes |
| Y Hu et al. | 2020 | Aquilion TSX-101A (Toshiba Medical Systems), Discovery 750 HD (GE), and Discovery VCT(GE) | Pre-treatment | Manually | NA | Yes |
| Z Yang et al. | 2019 | Brilliance CT Big Bore Oncology Configuration (Philips) | Pre-treatment | Manually | NA | Yes |
| J Zhu et al. | 2025 | SOMATOM Definition Flash (Siemens), Brilliance 64 (Philips), BrightSpeed (GE), Brilliance 16 (Philips), LightSpeed Ultra (GE), and Sensation 4 (Siemens) | Pre-treatment & post-treatment | Manually | Artery phase and venous phase | Yes |
| Y Sun et al. | 2024 | NA | Pre-treatment & post-treatment | Automatically | NA | Yes |
| Q Gu et al. | 2025 | Optima CT680(GE),  Definition Flash(Siemens-), Ingenuity CT(Philips) | Pre-treatment | Manually | NA | Yes |
| [X Ling](https://pubmed.ncbi.nlm.nih.gov/?term=) et al. | 2025 | NA(Siemens) | post-treatment | Manually | Venous phase | Yes |
| L Liu et al. | 2025 | Discovery CT750 HD (GE), AQUILION TSX-101A (Canon), SOMATOM Force (Siemens), Brilliance iCT (Philips), uCT 78 (United Imaging) | post-treatment | Manually | Artery phase | Yes |
| Y Liu et al. | 2025 | SOMATOM Definition AS (Siemens), Brilliance Big Bore (Philips), Big Bore (Philips), Discovery ST (GE), SOMATOM PLUS 4 (Siemens), Discovery 690 (GE), LightSpeed16 (GE), Discovery CT750 HD (GE), iCT 256 (Philips), Volume Zoom (Siemens),  Brilliance 64 (Philips), MX 4000 Dual (Philips),  uCT 510 (United Imaging), uCT S-160 (United Imaging), Ingenuity CT (Philips) | Pre-treatment | Manually | Venous phase | Yes |
| B Qi et al. | 2025 | BrightSpeed (GE), LightSpeed VCT (GE),  Optima CT680 Series (GE),  Optima CT680 Expert (GE),  Revolution EVO (GE),  SOMATOM Definition (Siemens),  SOMATOM Drive (Siemens),  IQon - Spectral (Philips) | Pre-treatment | Manually | NA | Yes |
| [L Shi](https://pubmed.ncbi.nlm.nih.gov/?term=) et al. | 2026 | NA | Pre-treatment & post-treatment | Manually | NA | Yes |
| Z Xu et al. | 2026 | NA | Pre-treatment | Manually | Venous phase | Yes |
| B Zhao et al. | 2026 | Lightspeed VCT (GE), Discovery 750HD (GE), Brilliance iCT (Philips) | Pre-treatment | Manually | Venous phase | Yes |
| Z Zhao et al. | 2025 | Discovery CT750 HD (GE), SOMATOM Force (Siemens), uCT 780 (United Imaging), IQon Spectral CT (Philips) | Pre-treatment | Manually | Venous phase | Yes |
| B Xu et al. | 2025 | SOMATOM Definition Flash (Siemens), Brilliance 64 (Philips) | Pre-treatment & post-treatment | Manually | NA | Yes |

### **Table S6.** Technical aspects of included studies for MRI.

| **Author** | **Year** | **Scanner Modality (Manufacturer)** | **Evaluation Time** | **Regions of interest** | **MRI sequence** | **Contrast-enhanced** |
| --- | --- | --- | --- | --- | --- | --- |
| Y Liu et al. | 2024 | Discovery MR750w 3.0T (GE), Discovery MR750 3.0T (GE), Skyra 3.0T (Siemens), Avanto 1.5T (Siemens), uMR780 3.0T (UIH), uMR588 1.5T (UIH) | Pre-treatment | Manually | T2WI | No |
| Y Liu et al. | 2024 | Discovery MR750w 3.0 T (GE), Discovery MR750 3.0 T (GE) | Pre-treatment | Manually | T2WI | No |
| A Hirata et al. | 2025 | Achieva 1.5 T Nova Dual (Philips) | Pre-treatment | Semi-automatically | DWI | No |

T1WI T1-weighted imaging; T2WI T2-weighted imaging.

### **Table S7.** Meta-regression of imaging-based artificial intelligence models for predicting pathological complete response in esophageal cancer in the internal validation set.

| **Subgroup** | **Number of studies, n** | **Sensitivity(95%CI****)** | **Meta-regression *P*-value** | **Specificity(95%CI)** | **Meta-regression *P*-value** |
| --- | --- | --- | --- | --- | --- |
| AI method |  |  | 0.01 |  | <0.001 |
| Machine learning | 21 | 0.77 (0.71 - 0.82) |  | 0.82 ( 0.77 - 0.86) |  |
| Deep learning | 6 | 0.77 (0.68 - 0.87) |  | 0.84 (0.77 - 0.91) |  |
| AI model |  |  | <0.001 |  | <0.001 |
| Radiomic & clinical model | 8 | 0.75 (0.67 - 0.84) |  | 0.84 (0.77 - 0.91) |  |
| Radiomic model | 19 | 0.78 (0.72 - 0.83) |  | 0.82 (0.77 - 0.86) |  |
| Contrast-enhanced |  |  | 0.13 |  | 0.11 |
| Yes | 23 | 0.75 (0.70 - 0.80) |  | 0.83 (0.78 - 0.87) |  |
| No | 2 | 0.82 (0.66 - 0.99) |  | 0.86 (0.72 - 0.99) |  |

### **Table S8.** Meta-regression of imaging-based artificial intelligence models for predicting pathological complete response in esophageal cancer in the external validation set.

| **Subgroup** | **Number of studies, n** | **Sensitivity(95%CI)** | **Meta-regression *P*-value** | **Specificity(95%CI)** | **Meta-regression *P*-value** |
| --- | --- | --- | --- | --- | --- |
| AI method |  |  | 0.13 |  | 0.97 |
| Machine learning | 12 | 0.70 (0.58 - 0.82) |  | 0.85 (0.76 - 0.93) |  |
| Deep learning | 4 | 0.80 (0.64 - 0.95) |  | 0.76 (0.56 - 0.97) |  |
| AI model |  |  | 0.81 |  | 0.37 |
| Radiomic & clinical model | 3 | 0.74 (0.47 - 1.00) |  | 0.93 (0.85 - 1.00) |  |
| Radiomic model | 13 | 0.73 (0.62 - 0.84) |  | 0.80 (0.70 - 0.89) |  |
| Contrast-enhanced |  |  | 0.83 |  | >.99 |
| Yes | 15 | 0.72 (0.62 - 0.83) |  | 0.83 (0.74 - 0.91) |  |
| No | 1 | 0.76 (0.42 - 1.00) |  | 0.86 (0.60 - 1.00) |  |

### **Table S9.** Selection of the first eligible imaging model reported in each article for the post hoc sensitivity analysis.

| **Study** | **Multiple models** | **Internal validation model** | **External validation model** | **Use in matched analysis** |
| --- | --- | --- | --- | --- |
| 1. J Wang et al. 2023 | Yes | tpCR imaging-only logistic regression (8 features) | Not reported | Internal: Replaced |
| 2. J Ou et al. 2024 | No | Baseline CT radiomics SVM | Baseline CT radiomics SVM | Internal: Unchanged; External: Unchanged |
| 3. X Wang et al. 2024 | Yes | Phase A pretherapeutic plain CT radiomics XGBoost | Not reported | Internal: Unavailable |
| 4. Z Zhang et al. 2025 | Yes | Vision-Mamba (CT plus voxel-level radiomics) | Vision-Mamba (CT plus voxel-level radiomics) | Internal: Unchanged; External: Unchanged |
| 5. Y Qi et al. 2025 | Yes | CT radiomics SVM | Not reported | Internal: Unchanged |
| 6. Y Zheng et al. 2024 | Yes | Not reported | Pretreatment CT radiomics LR | External: Replaced |
| 7. M Zhang et al. 2023 | Yes | RS1 pretreatment SVM | RS1 pretreatment SVM | Internal: Replaced; External: Replaced |
| 8. W-X Qi et al. 2024 | Yes | CT SVM | Not reported | Internal: Unchanged |
| 9. Z Lu et al. 2024 | Yes | Pure CT radiomics LR (Rad-score) | Not reported | Internal: Unavailable |
| 10. Y Liu et al. 2024 | Yes | Not reported | MRI radiomics RF | External: Unchanged |
| 11. Y Liu et al. 2024 | Yes | MRI radiomics RF | Not reported | Internal: Unavailable |
| 12. W Kong et al. 2024 | Yes | Not reported | Intratumoral Radiomics signature (RF) | External: Replaced |
| 13. L Fan et al. 2024 | Yes | Delta-radiomics signature (DRS) LR | Not reported | Internal: Unavailable |
| 14. Z Li et al. 2024 | Yes | Integrated-XGBoost | Integrated-XGBoost | Internal: Unavailable; External: Unavailable |
| 15. K Li et al. 2023 | Yes | Not reported | Radiomics ModelTLN SVM | External: Unavailable |
| 16. K Li et al. 2023 | Yes | Post-treatment SVM | Not reported | Internal: Replaced |
| 17. H Yue et al. 2022 | Yes | Cho2018 MRMR+RF | Not reported | Internal: Unavailable |
| 18. Y Li et al. 2021 | No | CT radiomics signature + clinical LR | Not reported | Internal: Unchanged |
| 19. Y Hu et al. 2020 | Yes | Not reported | Intratumoral SVM-RBF | External: Unavailable |
| 20. Z Yang et al. 2019 | Yes | Model1 LR bin32+wavelet | Not reported | Internal: Unchanged |
| 21. J Zhu et al. | Yes | DLRad1 | DLRad1 | Internal: Unchanged; External: Unchanged |
| 22. Y Sun et al. | Yes | LOMIA-T | Not reported | Internal: Unchanged |
| 23. Q Gu et al. | Yes | DL (VAE + FCN) | DL (VAE + FCN) | Internal: Unavailable; External: Unavailable |
| 24. A Hirata et al. | No | KNeighborsDist_BAG_L1 | Not reported | Internal: Unchanged |
| 25. X Ling et al. | Yes | Not reported | Combined-AdaBoost pCR | External: Unavailable |
| 26. L Liu et al. | Yes | RF-CT | Not reported | Internal: Unavailable |
| 27. Y Liu et al. | Yes | DELRN | DELRN | Internal: Unchanged; External: Unchanged |
| 28. B Qi et al. | Yes | Unimodal radiomics-XGBoost | Unimodal radiomics-XGBoost | Internal: Unchanged; External: Unchanged |
| 29. L Shi et al. | Yes | Radiomics | Not reported | Internal: Unavailable |
| 30. Z Xu et al. | Yes | Habitat-SVM | Habitat-SVM | Internal: Unavailable; External: Unavailable |
| 31. B Zhao et al. | Yes | Intratumoral radiomics score | Not reported | Internal: Unavailable |
| 32. Z Zhao et al. | Yes | MScaleCT | MScaleCT | Internal: Unchanged; External: Unchanged |
| 33. B Xu et al. | Yes | Delta-RFs signature | Not reported | Internal: Replaced |

Note: The selection rule was defined post hoc. Models were selected within the same validation cohort and pCR definition, following main-table number and displayed row or column order, or Results order if no eligible performance table was available. Pure clinical and conventional clinicoradiological comparator models were excluded. Unchanged indicates that the model and extracted counts were identical to those used in the primary analysis. Unavailable includes missing threshold data, cross-validation averages that could not yield a unique fourfold table, and incompatible reported denominators or metrics. Seven datasets from six publications changed model; 19 datasets used the same model in both analyses.

### **Table S10.** Comparison of the highest-AUC model and the first eligible imaging model reported in each article.

| **Validation** | **Analysis** | **Datasets, n** | **Sensitivity (95% CI)** | **Specificity (95% CI)** | **DOR (95% CI)** |
| --- | --- | --- | --- | --- | --- |
| Internal | All datasets: highest-AUC model | 27 | 0.77 (0.72 - 0.81) | 0.82 (0.78 - 0.86) | 15.49 (11.16 - 21.50) |
| Internal | Matched: highest-AUC model | 16 | 0.74 (0.66 - 0.80) | 0.80 (0.75 - 0.85) | 11.64 (7.62 - 17.78) |
| Internal | Matched: first eligible model | 16 | 0.73 (0.63 - 0.81) | 0.79 (0.73 - 0.85) | 10.16 (6.28 - 16.43) |
| External | All datasets: highest-AUC model | 16 | 0.73 (0.61 - 0.81) | 0.83 (0.73 - 0.90) | 12.93 (7.25 - 23.07) |
| External | Matched: highest-AUC model | 10 | 0.73 (0.65 - 0.80) | 0.77 (0.64 - 0.87) | 9.27 (4.66 - 18.43) |
| External | Matched: first eligible model | 10 | 0.72 (0.63 - 0.78) | 0.77 (0.61 - 0.87) | 8.20 (3.90 - 17.21) |

Note: Comparisons used identical datasets in each matched pair: internal validation, 16 datasets and 1,042 patients; external validation, ten datasets and 482 patients. The full original analysis is shown to distinguish data-availability effects from model-selection effects. Differences are descriptive, not tests of superiority. The external matched-subset fits estimated a boundary random-effects correlation; their results are exploratory. Counts for unchanged models were taken from the primary extraction; discrepancies between extracted counts and published results limited interpretation. CI, confidence interval; DOR, diagnostic odds ratio.

Model replacements in internal validation (four of 16 matched datasets): J Wang et al. (2023), M Zhang et al. (2023), K Li et al. (2023), and B Xu et al. (2025). The other 12 datasets retained their original models.

Model replacements in external validation (three of ten matched datasets): Y Zheng et al. (2024), M Zhang et al. (2023), and W Kong et al. (2024). The other seven datasets retained their original models.

These seven dataset-level replacements involved six publications; M Zhang et al. (2023) contributed replacements in both validation groups.

### **Figure S1.** Sensitivity and specificity forest plots. (A) Internal validation. Squares show individual-study estimates with exact binomial 95% confidence intervals; diamonds show pooled estimates and 95% confidence intervals. Separate red lines show marginal 95% prediction intervals. CI, confidence interval; PI, prediction interval. Outcome-specific I² describes inconsistency on the logit scale; Q, its degrees of freedom, and its *P* value are reported alongside it. The between-study variance (τ²) and standard deviation (τ) were derived from the bivariate model.

**Figure S1** (continued). (B) External validation. Symbols and intervals are defined on the preceding page.

### **Figure S2.** Summary receiver operating characteristic curves for internal (A) and external (B) validation. Points represent individual datasets and diamonds the pooled sensitivity and specificity. Dashed and dotted contours show joint 95% confidence and prediction regions, respectively. The separately reported marginal 95% prediction intervals describe sensitivity and specificity individually and are not projections of the joint contour.

### **Figure S3.** Forest plots of subgroup analyses across different artificial intelligence algorithms for predicting pathological complete response after neoadjuvant therapy in esophageal cancer in the internal (A) and external (B) validation sets*.*

### **Figure S4.** Forest plots of sensitivity analysis excluding outliers in bivariate box plots for imaging-based artificial intelligence models in the internal (A) and external (B) validation sets for predicting pathological complete response in esophageal cancer.

### **Figure S5.** Forest plots of sensitivity analyses excluding low-quality studies for imaging-based artificial intelligence models predicting pathological complete response in esophageal cancer in the internal (A) and external (B) validation sets*.*

### **Figure S6.** Forest plots of sensitivity analyses including only ESCC patients for the internal (A) and external (B) validation sets.
