# Supplementary material for: Imaging-Based AI for Predicting Pathological Complete Response in Esophageal Cancer After Neoadjuvant Therapy: Systematic Review and Meta-Analysis
Source: J Med Internet Res. 2026 Nov 11;28:e98520. doi: 10.2196/98520 (PMC13754179; doi:10.2196/98520)
Supplement: Supplementary file 3 [file jmir-v28-e98520-s003.docx]

### PRISMA 2020 for Abstracts Checklist

| **Section and Topic** | **Item #** | **Checklist item** | **Reported (Yes/No)** |
| --- | --- | --- | --- |
|  | | |  |
| TITLE | | |  |
| Title | 1 | Identify the report as a systematic review. | Yes (page 1) |
| BACKGROUND | | |  |
| Objectives | 2 | Provide an explicit statement of the main objective(s) or question(s) the review addresses. | Yes (page 1) |
| METHODS | | |  |
| Eligibility criteria | 3 | Specify the inclusion and exclusion criteria for the review. | Yes (page 1) |
| Information sources | 4 | Specify the information sources (e.g. databases, registers) used to identify studies and the date when each was last searched. | Yes (page 1) |
| Risk of bias | 5 | Specify the methods used to assess risk of bias in the included studies. | Yes (page 1) |
| Synthesis of results | 6 | Specify the methods used to present and synthesise results. | Yes (page 1) |
| RESULTS | | |  |
| Included studies | 7 | Give the total number of included studies and participants and summarise relevant characteristics of studies. | Yes (page 1) |
| Synthesis of results | 8 | Present results for main outcomes, preferably indicating the number of included studies and participants for each. If meta-analysis was done, report the summary estimate and confidence/credible interval. If comparing groups, indicate the direction of the effect (i.e. which group is favoured). | Yes (page 1) |
| DISCUSSION | | |  |
| Limitations of evidence | 9 | Provide a brief summary of the limitations of the evidence included in the review (e.g. study risk of bias, inconsistency and imprecision). | Yes (page 1) |
| Interpretation | 10 | Provide a general interpretation of the results and important implications. | Yes (page 1) |
| OTHER | | |  |
| Funding | 11 | Specify the primary source of funding for the review. | No (not in abstract; Funding section, page 16) |
| Registration | 12 | Provide the register name and registration number. | Yes (registration line, page 1) |
