# Supplementary material for: Imaging-Based AI for Predicting Pathological Complete Response in Esophageal Cancer After Neoadjuvant Therapy: Systematic Review and Meta-Analysis
Source: J Med Internet Res. 2026 Nov 11;28:e98520. doi: 10.2196/98520 (PMC13754179; doi:10.2196/98520)
Supplement: Supplementary file 4 [file jmir-v28-e98520-s004.docx]

### **PRISMA-S Checklist.**

| **Section/topic** | **#** | **Checklist item** | **Location(s) Reported** |
| --- | --- | --- | --- |
| INFORMATION SOURCES AND METHODS | | | |
| Database name | 1 | Name each individual database searched, stating the platform for each. | Methods (Information Sources and Search Strategy, pages 2–3); Table S1 in Multimedia Appendix 1 |
| Multi-database searching | 2 | If databases were searched simultaneously on a single platform, state the name of the platform, listing all of the databases searched. | Not applicable (each database was searched separately) |
| Study registries | 3 | List any study registries searched. | Not applicable (no study registries were searched) |
| Online resources and browsing | 4 | Describe any online or print source purposefully searched or browsed (e.g., tables of contents, print conference proceedings, web sites), and how this was done. | Not applicable (no online resources or browsing were used) |
| Citation searching | 5 | Indicate whether cited references or citing references were examined, and describe any methods used for locating cited/citing references (e.g., browsing reference lists, using a citation index, setting up email alerts for references citing included studies). | Methods (Information Sources and Search Strategy, pages 2–3: reference lists of included studies and similar systematic reviews and meta-analyses) |
| Contacts | 6 | Indicate whether additional studies or data were sought by contacting authors, experts, manufacturers, or others. | Methods (Data Collection Process and Data Items, page 3: corresponding authors were contacted for missing data) |
| Other methods | 7 | Describe any additional information sources or search methods used. | Methods (Information Sources and Search Strategy, pages 2–3: citation searching) |
| SEARCH STRATEGIES | | | |
| Full search strategies | 8 | Include the search strategies for each database and information source, copied and pasted exactly as run. | Table S1 in Multimedia Appendix 1 |
| Limits and restrictions | 9 | Specify that no limits were used, or describe any limits or restrictions applied to a search (e.g., date or time period, language, study design) and provide justification for their use. | Methods (Information Sources and Search Strategy, pages 2–3: no language or publication-year restrictions); Eligibility Criteria (page 3: English-language restriction) |
| Search filters | 10 | Indicate whether published search filters were used (as originally designed or modified), and if so, cite the filter(s) used. | Not applicable (no published search filters were used) |
| Prior work | 11 | Indicate when search strategies from other literature reviews were adapted or reused for a substantive part or all of the search, citing the previous review(s). | Not applicable (the search strategies were developed de novo for this review) |
| Updates | 12 | Report the methods used to update the search(es) (e.g., rerunning searches, email alerts). | Methods (Information Sources and Search Strategy, pages 2–3: updated search through August 7, 2026); Table S1 in Multimedia Appendix 1 |
| Dates of searches | 13 | For each search strategy, provide the date when the last search occurred. | Methods (Information Sources and Search Strategy, pages 2–3: last search August 7, 2026); Table S1 in Multimedia Appendix 1 |
| PEER REVIEW | | | |
| Peer review | 14 | Describe any search peer review process. | Not applicable (no formal peer review of the search strategy was performed) |
| MANAGING RECORDS | | | |
| Total Records | 15 | Document the total number of records identified from each database and other information sources. | Results (Study Selection, page 5: 1,680 database records and 113 records from citation searching); Table S1 in Multimedia Appendix 1 |
| Deduplication | 16 | Describe the processes and any software used to deduplicate records from multiple database searches and other information sources. | Results (Study Selection, page 5: 693 duplicate records removed using EndNote 20) |

PRISMA-S: An Extension to the PRISMA Statement for Reporting Literature Searches in Systematic Reviews

Rethlefsen ML, Kirtley S, Waffenschmidt S, Ayala AP, Moher D, Page MJ, Koffel JB, PRISMA-S Group.

Last updated February 27, 2020.
